# Acute Effects of Exercise Mode on Arterial Stiffness and Wave Reflection in Healthy Young Adults: A Systematic Review and Meta-Analysis

**DOI:** 10.3389/fphys.2018.00073

**Published:** 2018-02-13

**Authors:** Doris R. Pierce, Kenji Doma, Anthony S. Leicht

**Affiliations:** ^1^Sport and Exercise Science, James Cook University, Cairns, QLD, Australia; ^2^Sport and Exercise Science, James Cook University, Townsville, QLD, Australia

**Keywords:** carotid-femoral pulse wave velocity, augmentation index, aerobic exercise, resistance exercise, cardiovascular health

## Abstract

**Background:** This systematic review and meta-analysis quantified the effect of acute exercise mode on arterial stiffness and wave reflection measures including carotid-femoral pulse wave velocity (cf-PWV), augmentation index (AIx), and heart rate corrected AIx (AIx75).

**Methods:** Using standardized terms, database searches from inception until 2017 identified 45 studies. Eligible studies included acute aerobic and/or resistance exercise in healthy adults, pre- and post-intervention measurements or change values, and described their study design. Data from included studies were analyzed and reported in accordance with the Cochrane Handbook for Systematic Reviews of Interventions and PRISMA guidelines. Meta-analytical data were reported via forest plots using absolute differences with 95% confidence intervals with the random effects model accounting for between-study heterogeneity. Reporting bias was assessed via funnel plots and, individual studies were evaluated for bias using the Cochrane Collaboration's tool for assessing risk of bias. A modified PEDro Scale was applied to appraise methodological concerns inherent to included studies.

**Results:** Acute aerobic exercise failed to change cf-PWV (mean difference: 0.00 ms^−1^ [95% confidence interval: −0.11, 0.11], *p* = 0.96), significantly reduced AIx (−4.54% [−7.05, −2.04], *p* = 0.0004) and significantly increased AIx75 (3.58% [0.56, 6.61], *p* = 0.02). Contrastingly, acute resistance exercise significantly increased cf-PWV (0.42 ms^−1^ [0.17, 0.66], *p* = 0.0008), did not change AIx (1.63% [−3.83, 7.09], *p* = 0.56), and significantly increased AIx75 (15.02% [8.71, 21.33], *p* < 0.00001). Significant heterogeneity was evident within all comparisons except cf-PWV following resistance exercise, and several methodological concerns including low applicability of exercise protocols and lack of control intervention were identified.

**Conclusions:** Distinct arterial stiffness and wave reflection responses were identified following acute exercise with overall increases in both cf-PWV and AIx75 following resistance exercise potentially arising fromcardiovascular and non-cardiovascular factors that likely differ from those following aerobic exercise. Future studies should address identified methodological limitations to enhance interpretation and applicability of arterial stiffness and wave reflection indices to exercise and health.

## Introduction

Cardiovascular disease (CVD) is projected to remain the leading cause of death worldwide until 2030 with measures for early detection and progress monitoring essential to manage the condition and its associated health costs (Pereira et al., 2015). Arterial stiffness, typically reported from assessments of carotid-femoral pulse wave velocity (cf-PWV), has been validated as an independent predictor of future incidence of coronary heart disease, stroke, and mortality (Mattace-Raso et al., 2006) with greater arterial stiffness considered one of the first signs of pathological arterial wall modifications leading to CVD (Pereira et al., 2015). While arterial stiffness increases naturally with aging (Shirwany and Zou, 2010), augmented arterial stiffness has been associated with long-term, poor lifestyle choices such as inadequate diet, lack of physical activity, and smoking (Shirwany and Zou, 2010). Altered arterial stiffness has been implicated as a primary haemodynamic factor influencing augmentation index (AIx) (Kelly et al., 2001), a measure of wave reflection and left ventricular afterload, and diagnostically useful indicator of future CVD risk, particularly in younger individuals (McEniery et al., 2005). Arterial stiffening contributes to augmented cf-PWV and AIx, which increases cardiac workload and reduces coronary perfusion, thereby advancing the future risk of left ventricular hypertrophy and myocardial ischemia (Cavalcante et al., 2011). Subsequently, actions to minimize increases in arterial stiffness may be vital to manage CVD risk.

A recent systematic review and meta-analysis of 42 randomized, controlled trials reported that chronic aerobic training resulted in reduced cf-PWV and AIx, whereas chronic resistance and combined (aerobic + resistance) training had no effect on these measures (Ashor et al., 2014). A consequent review of 17 randomized, controlled trials also reported reductions in cf-PWV following aerobic but not resistance training (Li et al., 2015). Collectively, these reviews indicated exercise mode as an important modulator of chronic arterial stiffness and wave reflection responses.

While the effects of chronic exercise have been collated (Ashor et al., 2014; Li et al., 2015), no reviews to date have systematically examined the overall acute effects of exercise mode (i.e., aerobic vs. resistance exercise) on arterial stiffness and wave reflection. Numerous cross-group studies investigating the effects of acute exercise on these measures have produced inconclusive findings with some studies reporting decreases (Kingwell et al., 1997; Sugawara et al., 2015; Kobayashi et al., 2017), no change (Campbell et al., 2011; Ranadive et al., 2012; Ribeiro et al., 2014), or increases (Fahs et al., 2009; Yoon et al., 2010; Kingsley et al., 2016) in cf-PWV and/or AIx. Factors such as selection of outcome measures, timing of measurement, and, particularly, exercise mode, may explain the divergent findings. Findings from a review that will identify the acute responses to exercise modes will clarify common limitations of research designs, and provide recommendations to improve the experimental approach for future studies. Furthermore, such findings may identify the potential beneficial or adverse effects of acute exercise modes on arterial stiffness and wave reflection that may assist in understanding the impact of exercise on cardiovascular function for improved health. This systematic review and meta-analysis aimed to investigate and quantify the effect of exercise mode, during a single bout, on post-exercise cf-PWV, AIx, and AIx normalized to a heart rate of 75 beats per minute (AIx75). Since aerobic and resistance exercise modes are currently endorsed by major health organizations for comprehensive health programmes (Williams et al., 2007; Garber et al., 2011), findings from this review and meta-analysis may determine the potential exercise mode effects on arterial stiffness and wave reflection for cardiovascular health.

## Methods

This systematic review was conducted in accordance with the Cochrane Handbook for Systematic Reviews of Interventions (Higgins and Green, 2011) and reported in line with PRISMA guidelines (Liberati et al., 2009).

### Eligibility criteria

Studies were deemed eligible and included into the review if they examined all of the following criteria: (1) included apparently healthy young human adults ≤45 years; (2) investigated a single bout of aerobic and/or resistance exercise only or included a comparative intervention (e.g., no exercise); (3) included an exercise alone condition in studies with additional exposures (e.g., vascular occlusion); (4) reported pre- and post-intervention measurements or change values of cf-PWV, AIx, and/or AIx75 (aortic AIx only); and (5) described their study design. Exclusion criteria included studies that involved: (1) athletes or highly trained participants (e.g., ≥10 years of training history); (2) anaerobic exercise (e.g., Wingate) or isometric resistance exercise interventions; and (3) no pre- or post-intervention measurements. All studies were screened to ensure that participants had no pre-existing medical conditions that could affect arterial stiffness and wave reflection responses to exercise. To control for the potential influence of age, particularly older age, on these responses (Thiebaud et al., 2016), only studies with participants aged 18–45 years were included in this review. Studies with clinical populations were eligible for inclusion in this review if they incorporated a control group of healthy participants; only measurements for the healthy control group were reported and/or included in the analyses. Similarly, if the exercise intervention involved additional stressors (e.g., heat exposure), only the exercise alone condition was considered for analyses. Furthermore, studies had to report one or more of the following outcome measures, which are considered diagnostically superior predictors of future CVD risk (Davies and Struthers, 2003; Franklin, 2008): cf-PWV, AIx, or AIx75. Finally, only studies that focused on exercise modalities recommended for the maintenance and improvement of cardiovascular and general health (Garber et al., 2011) were considered for inclusion.

### Data sources and search strategies

Using standardized terms, a search of the PubMed, Ovid MEDLINE, Cochrane Library, and SPORTDiscus databases from database inception until the search date (10-16/05/2017) was conducted. Searches were limited to studies involving “Humans” and reported in English. Keywords used in the searches were: “arterial stiffness,” “vascular stiffness,” “acute exercise,” “pulse wave velocity,” “pulse wave analysis,” “augmentation index,” “PWV,” “PWA,” “AIx,” “AIx75.” The PubMed search strategy is detailed in the Supplementary Material (Supplementary Figure 1) and search strategies were adapted for each database. Additionally, reference lists of relevant articles and reviews identified in the searches were searched for eligible studies. Database alerts for recently published studies (May 2017–September 2017) were also continuously monitored for potentially eligible studies.

### Review of search outcomes

**Figure 1:**
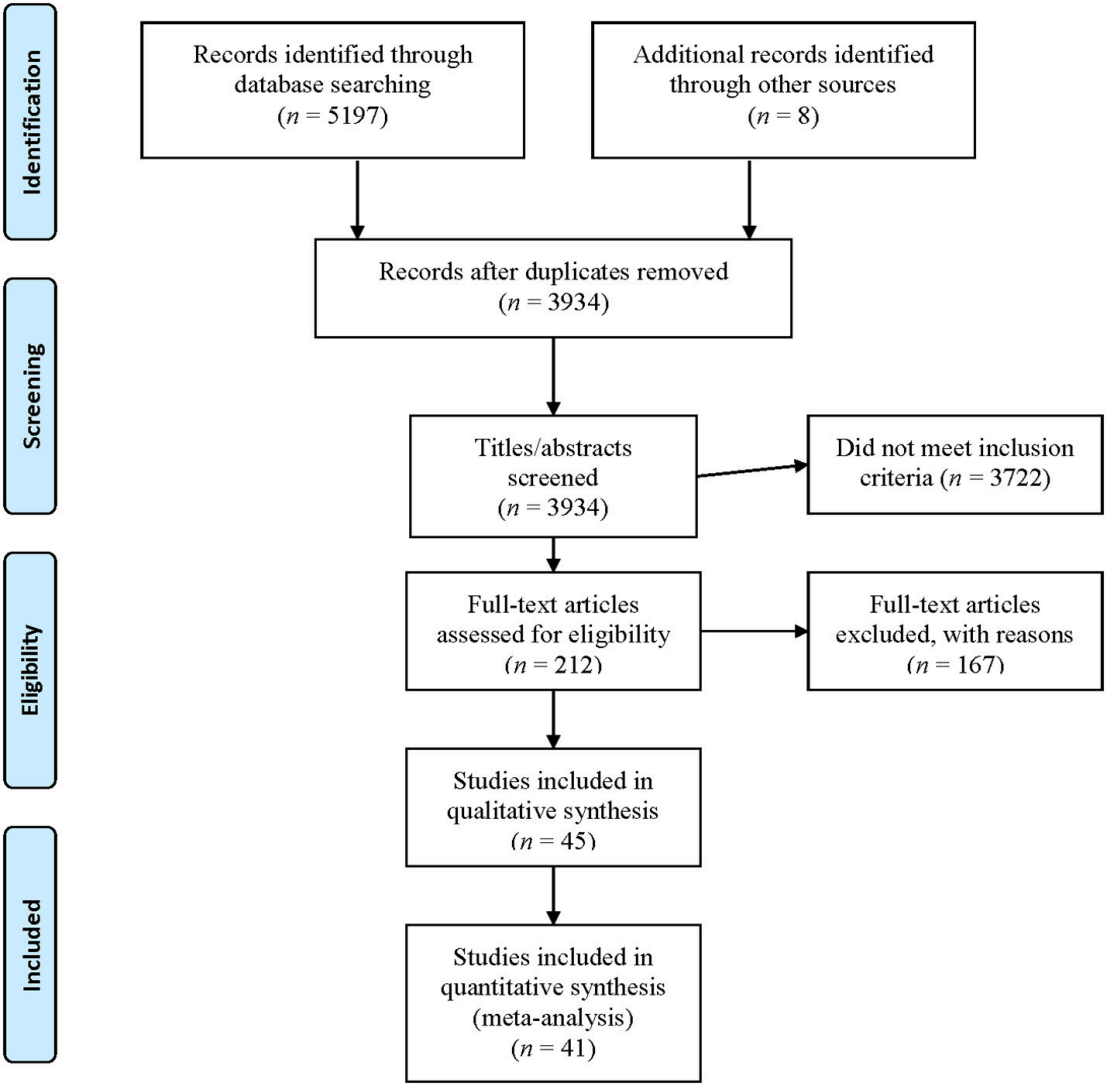
Selection process detailing the implemented search procedure in assessing eligibility for inclusion in this systematic review and meta-analysis.

One reviewer (DP) conducted the initial searches and screening of all identified titles, abstracts, and full original articles in line with the eligibility criteria (Figure 1). To assess the clarity of the inclusion/exclusion criteria, 212 articles were also independently screened by two other reviewers (ASL and KD) for inclusion into this review with initial inter-rater reliability >0.86 (McHugh, 2012). Where discrepancies between reviewers were present, decisions to include studies were discussed until consensus was reached. A total of 45 articles were identified for inclusion into this review (Figure 1). As no common post-intervention time point was assessed in all studies included in the review, data from the last time point within the immediate 60-min, post-intervention period were utilized for the meta-analysis. Three studies were excluded from the meta-analysis (Barnes et al., 2010; Lin et al., 2016; Perdomo et al., 2016) as they did not include data within the 60-min post-intervention period, while a fourth one (Collier et al., 2010) was excluded due to lack of pre-intervention data that could not be obtained from the corresponding author upon follow-up, leaving 41 studies to be included in the meta-analysis.

### Data extraction and quality assessment

A customized form was used to extract information about study design, participant details (e.g., health status, number, age, sex, stage of menstrual cycle in females, smoking status, activity level, recruitment strategy), study aim and hypothesis, methodology (e.g., intervention details, outcome measures, data collection time points, instruments/systems used), main findings, limitations, and conclusions. Results for each study's outcome measures were then entered into Microsoft Excel with pre- and post-intervention values for each outcome measure and time point entered as mean ± standard deviation (*SD*). For cross-over trials, mean ± *SD* were reported separately for each condition.

Methodological aspects were appraised using a modified PEDro scale. As blinding of participants or researchers to an exercise intervention was difficult, if not impossible, items 5–7 (i.e., blinding) of the original PEDro scale (Elkins et al., 2010) were replaced with items describing *a priori* power analysis, adequacy of intervention details, and inclusion of a control group. Two further items assessing bias (i.e., reporting of dropout/participation rates and sources of other potential bias) were added, resulting in a maximum quality score of 13. Additionally, the Cochrane Collaboration's tool for assessing risk of bias (i.e., selection, performance, detection, attrition, reporting and other bias) using a domain-based evaluation (Higgins and Green, 2011) was applied to all studies, as traditional scales and checklists generally contain items that are not directly related to internal validity (Higgins and Green, 2011).

Corresponding authors were contacted where data were not clear or unavailable, and data were updated for inclusion into this review. Several studies investigating the acute effects of aerobic exercise were included in a previous review of acute aerobic exercise (Mutter et al., 2016), and data extracted were cross-referenced with the previously reported data for quality assurance, where available.

Reporting bias, including publication bias, was minimized by use of eligibility criteria, consensus of multiple reviewers, and inspection of funnel plots. Funnel plots are simple scatter plots with the studies' mean differences plotted on the x-axis and the standard error on the y-axis (Sterne and Egger, 2001). In the absence of publication bias, the plots should resemble an inverted funnel (Sterne and Egger, 2001). Participant selection bias was inherent within the incorporated studies, as only studies investigating healthy, young adult participants were included. However, this was intentional, as the aim was to review normal arterial stiffness and wave reflection responses after different modes of acute exercise in this healthy population group.

### Statistical analysis

Forest and funnel plots were generated using Review Manager Software (Review Manager [RevMan]. Version 5.3. Copenhagen: The Nordic Cochrane Centre, The Cochrane Collaboration, 2014), and data are presented as mean ± *SD*, unless otherwise stated. Data originally reported as mean ± standard error or mean ± confidence interval were converted to mean ± *SD* for consistency following Cochrane guidelines (Higgins and Green, 2011). Where only baseline and change values (post-pre) were reported in a study, the post-intervention measurement was computed by adding the means and calculating the pooled *SD* (Higgins and Green, 2011). Data not reported in tables or the main text were extracted manually from figures as described previously (Kadic et al., 2016). The outcome of each individual meta-analysis represents the pre-post intervention difference in outcome variable (i.e., cf-PWV, AIx, or AIx75) for each intervention. The meta-analytical data were reported via forest plots using absolute differences with 95% confidence interval, given that each outcome measure (i.e., cf-PWV, AIx, and AIx75) was clustered separately (Higgins and Green, 2011). The random effects model was used to account for possible between-study heterogeneity regarding study design, participant characteristics, and methodology used to assess arterial stiffness and wave reflection (Higgins and Green, 2011). The percentage of variation across studies indicative of heterogeneity was reported using the *I*^2^ statistic and the Chi-squared test. Interpretation of the *I*^2^ statistic was in accordance with Cochrane guidelines as following: <40% might not be important; 30–60% may represent moderate heterogeneity; 50–90% may represent substantial heterogeneity; and >75% considerable heterogeneity (Higgins and Green, 2011). Furthermore, a statistically significant effect based on the Chi-squared test suggested evidence of heterogeneity (Higgins and Green, 2011). Pearson product-moment correlation coefficients were computed to assess the relationship between timing of post-intervention measurement and reported change (%) in cf-PWV, AIx, and AIx75. Statistical significance was determined with an alpha level of ≤0.05.

## Results

A total of 45 and 41 studies met the eligibility criteria and were included into the qualitative review and meta-analysis, respectively. Figure 1 summarizes the screening and selection process. Primary reasons for exclusion of articles during screening of the full-text records were assessment of arterial stiffness and wave reflection following chronic exercise training as opposed to acute exercise (92 studies) and reporting of outcome measures not aligned with our selection criteria (34 studies, Figure 1).

### Participants

The total number of participants across all studies was 1,211 (889 men, 297 women, 25 not specified) with a mean sample size of 22.4 ± 17.2 (range 9–122) and participant age ranging from 18 to 45 years. Twenty-six studies included men only, no study included women only, 18 included both men and women, and sex distribution was not reported for one study (Table 1). Participants' baseline physical activity status, although not reported in all studies, ranged from sedentary to highly physically active. Nine studies (Sharman et al., 2008; Fahs et al., 2009; Collier et al., 2010; Gkaliagkousi et al., 2014; Hanssen et al., 2015; Kingsley et al., 2016; Perdomo et al., 2016; Thiebaud et al., 2016; Tai et al., 2018) reported an *a priori* sample size calculation.

**Table 1:** Participant characteristics of included studies.

**Study**	**Participants**	***n***	**Age (years)**	**BMI (kg m^−2^)**	**Physical activity level**
Barnes et al., 2010	Healthy young men (P1, *n =* 11; P2, *n =* 16)	27	**P1** 25 ± 5.2 **P2** 24 ± 8 (18–38)	**P1** 25 ± 5.2 **P2** 23 ± 4	<2 days of exercise/week
Boutcher et al., 2011	Healthy young men with no family history of hypertension (NFHT, *n =* 20) and family history of hypertension (FHT, *n =* 20)	40	**NFHT** 21.3 ± 2.7 **FHT** 22.3 ± 2.2 (18–27)	**NFHT** 22.2 ± 2.2 **FHT** 21.9 ± 3.1	Both groups: moderate-intensity exercise > 30 min, 1–3 times/week for >2 years
Burr et al., 2015	Healthy young men (*n =* 9) and women (*n =* 4)	13	25 ± 6	H: 175 ± 8 cm BM: 74.6 ±16 kg (BMI not reported)	Recreationally active, not currently or previously engaged in eccentric exercise training
Campbell et al., 2011	Healthy young men	10	31 ± 5	24 ± 3	Not participating in regular vigorous exercise
Chandrakumar et al., 2015	Healthy young men	15	20.2 ± 0.8	21.8 ± 1.4	Not participating in aerobic exercise of >30 min duration more than 3 times/week
Collier et al., 2010	Healthy young men	10	24.9 ± 2.7	H: 175.8 ± 1.48 cm BM: 76.8 ± 2.36 kg (BMI not reported)	Moderately active
Doonan et al., 2011	Healthy young non-smoking (*n =* 53)	53	23 ± 5.4	22.3 ± 2.2	Not reported
Doonan et al., 2013	Healthy young men (M, *n =* 67) and women (W, *n =* 55), women in early follicular phase	122	**M** 24.4 ± 6.2 **W** 23.7 ± 4.8	**M** 22.8 ± 2.7 **W** 21.7 ± 2.1	Low PA (M/W): 12/13 Moderate PA (M/W): 33/26 High PA (M/W): 22/16
Fahs et al., 2009	Healthy young men	17	24.2 ± 2.9	27.0 ± 4.9	Not reported
Figueroa and Vicil, 2011	Healthy young men (*n =* 11) and women (*n =* 12), women in follicular phase	23	22 ± 2	23 ± 2	Physically active but not competitive athletes
Gkaliagkousi et al., 2014	Healthy men (*n =* 9) and women (*n =* 6)	15	39.3 ± 5.6	23.3 ± 2.8	Not reported
Hanssen et al., 2015	Healthy young men	21	19–31	23 ± 1	Not reported
Heffernan et al., 2006	Healthy young men (*n =* 7) and women (*n =* 6), women in early follicular phase	13	21.5 ± 2.5	25.7 ± 4.7	Not reported
Heffernan et al., 2007d	Healthy young men	13	25 ± 2.5 (21–29)	H: 174.7 ± 4.7 cm BM: 74.5 ± 7.9 (BMI not reported)	Moderately active
Heffernan et al., 2007b	Healthy young, resistance-trained (RT, *n =* 15) and non-resistance-trained (NRT, *n =* 15) men	30	**RT** 21.9 ± 2.3 **NRT** 22.7 ± 3.5	**RT** H: 177.9 ± 6.2 cm BM: 92.9 ± 12.8 kg **NRT** H: 179.1 ± 1.4 W: 81.6 ± 13.2 kg (BMI not reported)	3 days/week for 7.2 ± 3.3 years, aerobic exercise <1.5 h/week Sedentary/ recreationally active
Heffernan et al., 2007c	Healthy young men	14	27.9 ± 7.5	H: 180.6 ± 7.1 cm BM: 85.4 ± 15.7 kg (BMI not reported)	Not reported
Heffernan et al., 2007a	Healthy young African-American (AA, *n =* 12) and White (WH, *n =* 12) men	24	**AA** 22 ± 3.5 **WH** 22 ± 3.5	**AA** 27.3 ± 4.2 **WH** 28.7 ± 3.5	Not reported
Hu et al., 2013	Healthy young men (*n =* 10) and women (*n =* 5)	15	26.2 ± 2.3	23.9 ± 3.5	Sedentary/moderately active
Hull et al., 2011	Healthy young men (*n =* 18) and women (*n =* 7)	25	29.3 ± 5.8	23.1 ± 1.8	Sedentary/recreationally active
Kingsley et al., 2016	Healthy young men (*n =* 11) and women (*n =* 5), women in follicular phase	16	23 ± 3	H: 1.74 ± 0.11 m BM: 82.2 ± 17.7 kg (BMI not reported)	Resistance training ≥3 days/week for ≥2 years
Kingwell et al., 1997	Healthy young men	12	24 ± 6	22.9 ± 3.1	Sedentary
Kobayashi et al., 2017	Healthy young men	11	23.4 ± 1.9	21.5 ± 1.7	Sedentary (≥2 years without regular exercise)
Lane et al., 2013	Healthy young men (M, *n =* 31) and women (W, *n =* 31), women in early follicular phase or during oral contraceptive placebo phase	62	**M** 24.7 ± 3.3 **W** 24.8 ± 3.3 **ALL** 24.7 ± 3.1 (18–35)	**M** 26.0 ± 3.3 **W** 25.0 ± 4.5 **ALL** 25.6 ± 4.2	Sedentary (>6 months no structured exercise activity of any kind lasting longer than 30 min more than once/week)
Lefferts et al., 2015	Healthy young men	12	22 ± 3	24.6 ± 2.8	Physically active
Lin et al., 2016	Healthy young men	11	24 ± 4.9	23 ± 4.8	Sedentary or recreationally active but not participating in any type of resistance or endurance training
Lydakis et al., 2008	Healthy young men (*n =* 7) and women (*n =* 8)	15	26.6 ± 3.6	24.3 ± 3.1	Not reported
Mak and Lai, 2015	Healthy young men	18	21 ± 1 (20–24)	H: 169 ± 6 cm BM: 56.0 ± 7.5 kg	Not reported
Melo et al., 2016	Healthy young men (*n =* 24) and women (*n =* 21)	45	25.22 ± 6 (18–36)	BF: 21.62 ± 6.5% (BMI/Weight not reported)	Non-athletes
Milatz et al., 2015	Healthy men	32	33.7 ± 8	24.0 ± 2	Recreationally active (moderate aerobic activity ≥2 times/week for 60 min)
Moore et al., 2016	Healthy young men	34	21.53 ± 3	22.68 ± 1.6	Resistance training ≥3 times/week lasting at least 45 min/session
Munir et al., 2008	Healthy young adults (male or female not reported)	25	19–33	Physical characteristics not reported	Recreationally active
Perdomo et al., 2016	Healthy young men (M, *n =* 15) and women (W, *n =* 15)	30	**M** 23.4 ± 1.8 **W** 24.3 ± 3.0 **ALL** 23.8 ± 2.5	**M** 23.9 ± 1.7 **W** 23.4 ± 2.6 **ALL** 23.7 ± 2.2	Not reported
Peres et al., 2010	Healthy young men (*n =* 9) and women (*n =* 9)	18	20 ± 5	21.28 ± 2.63	Sedentary
Ranadive et al., 2012	Healthy young men (*n =* 9) and women (*n =* 6)	15	25 ± 5 (18–45)	22.9 ± 3.4	Not reported
Ribeiro et al., 2014	Healthy young men	14	31.0 ± 3.7	26.6 ± 3.4	Non-athletes
Sharman et al., 2008	Healthy young men	12	29 ± 3.5	23.8 ± 3.1	Not reported
Siasos et al., 2016a	Healthy young men	20	22.6 ± 3.3	Not reported	Not reported
Siasos et al., 2016b	Healthy young men	20	22.6 ± 3.3	22.03 ± 1.6	Not reported
Sugawara et al., 2015	Healthy young men	23	22 ± 4	21.9 ± 1.8	Not reported
Sun et al., 2015	Healthy Caucasian (CA) men and women (*n =* 15/15) and Chinese (CH) men and women (*n =* 16/16)	62	**CA** 24 ± 4 **CH** 28 ± 4	**CA** 23.1 ± 2.5 **CH** 22.5 ± 2.6	Sedentary
Tai et al., 2018	Healthy young men (*n =* 12) and women (*n =* 3)	15	23 ± 3 (18–28)	H: 1.74 ± 0.11 m BM: 82.2 ± 17.7 kg (BMI not reported)	Resistance training ≥ 3 days/week for ≥ 2 years
Thiebaud et al., 2016	Healthy young men (YG, *n =* 12)	12	**YG** 26.5 ± 3.3 (20–39)	**YG** 24.3 ± 1.8	Recreationally active
Yan et al., 2014	Healthy young African-American men (AAM, *n =* 28) and women (AAW, *n =* 24) and Caucasian men (CAM, *n =* 25) and women (CAW, *n =* 23)	100	**AAM** 25 ± 5.3 **AAW** 24 ± 4.9 **CAM** 25 ± 5 **CAW** 25 ± 4.8	**AAM** 26.7 ± 5.8 **AAW** 28.9 ± 5.4 **CAM** 25.7 ± 5.5 **CAW** 23.1 ± 5.	Not reported
Yan et al., 2017	Healthy young African-American men (*n =* 9) and women (*n =* 13) and Caucasian men (*n =* 14) and women (*n =* 13)	49	**AA** 23 ± 4.7 **CA** 21 ± 5.2	**AA** 25.1 ± 3.8 **CA** 24.2 ± 2.6	Most recreationally active
Yoon et al., 2010	Healthy young men	13	20.8 ± 2.2 (20–29)	23.4 ± 1.9	Resistance exercise ~3 times/week

*Data are presented as mean ± SD (range). BMI, body mass index; P1, protocol 1 (bilateral, eccentric-only leg press and sham control); P2, protocol 2 (unilateral, eccentric-only elbow flexion); NFHT, no family history of hypertension; FHT, family history of hypertension; H, height; BM, body mass; M, men, W, women; PA, physical activity; RT, resistance-trained; NRT, not resistance-trained; AA, African-American; WH, White; BF, body fat; CA, Caucasian; CH, Chinese; YG, young group; AAM, African-American men; AAW, African-American women; CAM, Caucasian men; CAW, Caucasian women*.

### Characteristics of studies within review

The study designs were variable and included 17 cross-over studies (Kingwell et al., 1997; Heffernan et al., 2007c,d; Barnes et al., 2010; Collier et al., 2010; Yoon et al., 2010; Figueroa and Vicil, 2011; Ranadive et al., 2012; Ribeiro et al., 2014; Hanssen et al., 2015; Lefferts et al., 2015; Siasos et al., 2016a,b; Thiebaud et al., 2016; Kobayashi et al., 2017; Yan et al., 2017; Tai et al., 2018), 24 pre-post interventions (Heffernan et al., 2006, 2007a,b; Lydakis et al., 2008; Munir et al., 2008; Peres et al., 2010; Boutcher et al., 2011; Doonan et al., 2011, 2013; Hull et al., 2011; Hu et al., 2013; Lane et al., 2013; Gkaliagkousi et al., 2014; Yan et al., 2014; Burr et al., 2015; Chandrakumar et al., 2015; Mak and Lai, 2015; Milatz et al., 2015; Sugawara et al., 2015; Sun et al., 2015; Kingsley et al., 2016; Melo et al., 2016; Moore et al., 2016; Perdomo et al., 2016) and four randomized, controlled trials utilizing a pharmaceutical/placebo design (Sharman et al., 2008; Fahs et al., 2009; Campbell et al., 2011; Lin et al., 2016). Thirty-four studies assessed the effects of aerobic exercise (Table 2) and 13 examined the effects of resistance exercise (Table 3); two of these studies (Heffernan et al., 2007d; Collier et al., 2010) compared the effects of aerobic vs. resistance exercise. Eight of the 45 studies (six resistance, two aerobic) included a control group that completed no exercise (Table 4). Aerobic exercise was either performed on a cycle ergometer (21 studies) or a treadmill (13 studies) with varying intensities and durations (Table 2).

**Table 2:** Summary of studies that examined carotid-femoral pulse wave velocity and/or augmentation indices post aerobic exercise intervention.

**Study**	**Participants**	**Exercise intervention**	**Duration**	**Intensity**	**Assessment time points; Method used**	Results (Group/time differences)^*^	**Quality score**
Boutcher et al., 2011	Healthy young men with no family history of HT (*n =* 20) and family history of HT (*n =* 20)	Cycling Ergometer	20 min	60% VO_2max_	Rest & 30 min post; SphygmoCor/AT	**AIx** No group and rest-post differences	9
Burr et al., 2015	Healthy young men (*n =* 9) and women (*n =* 4)	Treadmill running	40 min, 5 min active recovery	12% decline, 60% VO_2max_	Rest & 10 min, 6, 24, 48, and 72 h post; SphygmoCor/AT	**cf-PWV** Greater at 48 and 72 h post **AIx75** No rest-post differences	9
Campbell et al., 2011	Healthy young men (*n =* 10)	Cycling Ergometer	To volitional exhaustion after 3 min warm-up	Warm-up at 60 W at 60 rpm, then increasing by 30 W min^−1^	Rest & 0–5 min, 6–10 min and 11–15 min post; SphygmoCor/AT	**cf-PWV** No rest-post differences **AIx** Lower at all-time points post	10
Chandrakumar et al., 2015	Healthy young overweight (OW, *n =* 15) and healthy weight (HW, *n =* 15) men	Cycling Ergometer	30 min	65% VO_2max_ at 60–80 rpm	*cf-PWV* Rest & 60 min post *AIx* Rest & 10, 20, 30, and 60 min post; SphygmoCor 2000/AT	**cf-PWV** No group and rest-post differences**AIx**Greater in OW at all-time points	8
Collier et al., 2010	Healthy young men (*n =* 10)	Cycling Ergometer	30 min	65% VO_2peak_	Rest & 40 min and 60 min post;Doppler probes, ECG, BioPac MP100	**cf-PWV**No rest-post differences	10
Doonan et al., 2011	Healthy young non-smoking men (*n =* 53)	Treadmill running	To volitional exhaustion	Bruce protocol	Rest & 2, 5, 10, and 15 min post; SphygmoCor/AT	**cf-PWV** Greater at 2 and 5 min post **AIx75** Greater at each time point post	9
Doonan et al., 2013	Healthy young men (*n =* 67) and women (*n =* 55), women in early follicular phase	Treadmill running	To volitional exhaustion	Bruce protocol	Rest & 2, 5, 10 and 15 min post; SphygmoCor/AT	**cf-PWV**Greater for M and W at 2 min post Greater for M at all-time points**AIx75** No rest-post differences for M and W Greater for M and W at 5 min post	8
Gkaliagkousi et al., 2014	Healthy men (*n =* 9) and women (*n =* 6)	Treadmill running	To volitional exhaustion	Bruce protocol	Rest & 10, 30 and 60 min post; SphygmoCor/AT	**cf-PWV** No rest-post differences	9
Hanssen et al., 2015	Healthy young men (*n =* 21)	Treadmill running	**HIIT** 10 min warm-up, 4 × 4 min bouts, 3 min recovery **MCT** Duration computed to match HIIT workload	**HIIT** 70% HR_max_ 90–95% HR_max_ 70% HR_max_ **MCT** 80% HR_max_ (1% incline for both protocols)	Rest & 5, 20, 35, and 50 min post, every 2 h for 24 h post; SphygmoCor/AT (rest- and post- measurements) Mobil-O-Graph (24 h ambulatory monitoring)	**AIx**No rest-post differences Lower after HIIT compared to MCT at 35 and 50 min post **AIx75**Greater after HIIT compared to MCT at 5, 20 and 35 min post Greater at 5 and 20 min post after HIIT and at 5 min post after MCT 24 h post: lower after HIIT but not MCT	10
Heffernan et al., 2007d	Healthy young men (*n =* 13)	Cycling Ergometer	30 min	65% VO_2peak_	Rest & 20 min post; Doppler probes, ECG, BioPac MP100	**cf-PWV** Lower at 20 min post	9
Heffernan et al., 2007b	Healthy young resistance-trained (RT, *n =* 15) and not resistance-trained (NRT, *n =* 15) men	Cycling Ergometer	To volitional exhaustion	First workload at 50 W, then increased by 30 W every 2 min	Rest & 10, 20, and 30 min post; SphygmoCor/AT	**cf-PWV** No rest-post differences **AIx** Lower at 20 and 30 min post for both RT and NRT **AIx75** Greater at each time point post for both RT and NRT	9
Heffernan et al., 2007c	Healthy young African-American (AA, *n =* 12) and White (WH, *n =* 12) men	Cycling Ergometer	To volitional exhaustion	First workload 50 W, then increased by 30 W every 2 min	Rest & 15 and 30 min post; SphygmoCor/AT	**cf-PWV** Greater for AA compared to WH at all-time points No rest-post differences	7
Hu et al., 2013	Healthy young men (*n =* 10) and women (*n =* 5)	Cycling Ergometer	To volitional exhaustion	First workload 50 W, then increased by 30 W every 2 min	Rest & 3 min post; SphygmoCor/AT	**AIx** No rest-post differences **AIx75** Greater at 3 min post	8
Hull et al., 2011	Healthy young men (*n =* 18) and women (*n =* 7)	Cycling Ergometer	10 min	60% of age-predicted HR_max_ (50–60 rpm)	Rest & 0 min post; SphygmoCor/AT	**cf-PWV** Greater at 0 min post **AIx75** No rest-post differences	8
Kingwell et al., 1997	Healthy young men (*n =* 12)	Cycling Ergometer	30 min	65% VO_2max_	Rest & 30 and 60 min post; Custom-built software/AT	**cf-PWV** Lower at 30 min post	9
Kobayashi et al., 2017	Healthy young men (*n =* 11)	Cycling Ergometer	15, 30, and 45 min	65% VO_2peak_	Rest & 30, 60, and 90 min post; Calculation/AT	**cf-PWV** Lower at 30 min post after 15, 30 and 45 min exercise bouts Lower at 60 min post after 30 and 45 min exercise bouts	9
Lane et al., 2013	Healthy young men (*n =* 31) and women (*n =* 31)	Cycling Ergometer	To volitional exhaustion	First workload at 50 W, then increased by 30 W every 2 min	Rest & 15 and 30 min post; SphygmoCor/AT	**cf-PWV** Lower at 15 min post for W and at 30 min post for M **AIx** Lower at 15 and 30 min post for M and at 30 min post for W	8
Lefferts et al., 2015	Healthy young men (*n =* 12)	Treadmill walking	3 × 20 min bouts with 20 min rest between bouts (100 min total)	5% incline, ≈ 40% VO_2max_ (75–80% HR_max_)	Rest & 15–30 min post; SphygmoCor/AT	**cf-PWV** No rest-post differences	9
Lin et al., 2016	Healthy young men (*n =* 11)	Treadmill running	30 min	10% decline, 75% VO_2peak_	Rest & 90 min, 24, 48 and 72 h post; Millar Inc-Biopac/AT	**cf-PWV** Greater at 24 h post	10
Melo et al., 2016	Healthy young men (*n =* 24) and women (*n =* 21)	Treadmill running	To volitional exhaustion	Started at self-selected pace, then increments of 1 mph every 2 min for 4 min followed by 2.5% increase in grade every min	Rest & 10 min post; Complior/AT	**cf-PWV** No rest-post differences	7
Milatz et al., 2015	Healthy men (*n =* 32)	Cycling Ergometer	60 min	45% VO_2max_	Rest & 1, 15, 30, 45, and 60 min post; Mobil-O-Graph	**cf-PWV** Lower at 60 min post	8
Moore et al., 2016	Healthy overweight (OW, *n =* 17) and healthy-weight (HW, *n =* 17) men	Treadmill running	To volitional exhaustion	3-min progressive speed and grade stages	Rest & 2, 5, 10, 20, 30, 45, and 60 min post; SphygmoCor/AT	**cf-PWV** Greater in OW compared to HW at all-time points post	8
Munir et al., 2008	Healthy young adults (*n* = 25, male or female not reported)	Cycling Ergometer	12 min or to volitional exhaustion	Start at 25 W, increased by 25 W in 2 min intervals to a max. of 150 W	Rest & 1–3, 15, 30 and 60 min post; SphygmoCor/AT	**cf-PWV** No rest-post differences **AIx** Lower at 15, 30 and 60 min post	5
Perdomo et al., 2016	Healthy young men (*n =* 15) and women (*n =* 15)	Treadmill running	30 min	70–75% of age-predicted HR_max_	Rest & 24 h post; Complior Analyse/piezoelectric sensors	**cf-PWV** Greater in M than W at rest Lower at 24 h post in M only	8
Peres et al., 2010	Healthy young men (*n =* 9) and women (*n =* 9)	Cycling Ergometer	14 min or signs and symptoms of dyspnea, exhaustion, fatigue, myocardial ischemia or BP ≥160/100	Load increase every 2 min (60 rpm)	Rest & 0 min post; Complior/AT	**cf-PWV** Greater at immediately post	8
Ranadive et al., 2012	Healthy young men (*n =* 9) and women (*n =* 6)	Arm vs. leg cycling Ergometer	To volitional exhaustion	Leg: Start at 50 W, then 30 W increase every 2 min Arm: Start at 15 W and 15 W increases every 2 min	Rest & 10 min post; SphygmoCor/AT	**cf-PWV** No rest-post differences	9
Ribeiro et al., 2014	Healthy young men (*n =* 14)	Treadmill walking	10 min	5 km h^−1^	Rest & 0 min post; SphygmoCor SCOR-PX/AT	**AIx75** No rest-post differences	9
Sharman et al., 2008	Healthy young men (*n =* 12)	Cycling Ergometer	To reach 10-min period at steady-state HR	60% HR_max_	Rest & 2 and 10 min post; SphygmoCor version 7.01/AT	**AIx** No rest-post differences	12
Siasos et al., 2016a	Healthy young men (*n =* 20)	Cycling Ergometer	**HIAE** 30 × 30 s bouts with 1:1 work-rest ratio **CAE** 30 min	**HIAE** 100% max. aerobic capacity **CAE** 50% VO_2max_	Rest & 10 min post; SphygmoCor/AT	**AIx75** Lower after CAE but not HIAE	7
Siasos et al., 2016b	Healthy young men (*n =* 20)	Cycling Ergometer	**HIAE** 30 × 30 s bouts with 1:1 work-rest ratio **CAE** 30 min	**HIAE** 100% max. aerobic capacity **CAE** 50% VO_2max_	Rest & 10 min post; SphygmoCor/AT	**cf-PWV** No rest-post differences	8
Sugawara et al., 2015	Healthy young men (*n =* 23)	Cycling Ergometer	50 min	Warm-up: 65% HRR, thereafter 65–75% HRR	Rest & 20 and 50 min post; Omron-Colin/AT	**cf-PWV** Lower at 20 and 50 min post-intervention	7
Sun et al., 2015	Healthy Caucasian men and women (*n =* 15/15) and Chinese men and women (*n =* 16/16)	Treadmill running	45 min	70% HRR	Rest & 30 and 60 min post; SphygmoCor/AT	**cf-PWV** No rest-post and race differences	6
Yan et al., 2014	Healthy young African-American men (AAM, *n =* 28) and women (AAW, *n =* 24) and Caucasian men (CAM, *n =* 25) and women (CAW, *n =* 23)	Cycling Ergometer	To volitional exhaustion	First workload 50 W, then increased by 30 W every 2 min	Rest & 15 and 30 min post; SphygmoCor/AT	**cf-PWV** Lower in W compared to M at rest Greater at 30 min post in AA but lower in CA **AIx** Change in W greater than change in M **AIx75** Change in W different to change in M	7
Yan et al., 2017	Healthy young AAM and AAW (*n =* 9/13) and CAM and CAW (*n =* 14/13)	Treadmill running	45 min	70% HRR	Rest & 30, 60, and 90 min post; SphygmoCor/AT	**cf-PWV** No rest-post differences	11

*Data are presented as mean ± SD. AT, applanation tonometry; cf-PWV, carotid-femoral pulse wave velocity; HT, hypertension; VO_2max_, maximal oxygen consumption; AIx, augmentation index; AIx75, augmentation index corrected for heart rate of 75 beats per minute; OW, overweight; HW, healthy weight; VO_2peak_, peak oxygen consumption; ECG, electrocardiogram; M, men; W, women; HIIT, high-intensity interval training; MCT, moderate continuous training; HR_max_, maximal heart rate; RT, resistance-trained; NRT, not resistance-trained; AA, African-American; WH, white; rpm, rounds per minute; BP, blood pressure; HIAE, high-intensity aerobic exercise; CAE, continuous aerobic exercise; HRR, heart rate reserve*.

* *Differences reported at a statistically significant level (p ≤ 0.05)*.

**Table 3:** Summary of studies that examined carotid-femoral pulse wave velocity and/or augmentation indices post resistance exercise intervention.

**Study**	**Participants**	**Exercise intervention**	**Sets and repetitions**	**Intensity**	**Assessment time points; Method used**	Results (Group/time differences)^*^	**Quality score**
Barnes et al., 2010	Healthy young men (P1, *n =* 11; P2, *n =* 16)	**P1** bilateral, eccentric-only inclined leg press **P2** unilateral, eccentric-only elbow flexion on isokinetic dynamometer	**P1** 6 × 10 reps, 3–4 min between sets, **P2** 2 × 20 reps, 3 s per contraction, 4 min between sets	**P1** 110% of 1-RM **P2** not specified	Rest & 90 min, 24 48 and 72 h post; Omron-Colin VP2000/AT	**cf-PWV** Greater at 48 h post after P1 and P2	8
Collier et al., 2010	Healthy young men (*n =* 10)	Bench press, bent-over row, leg extension, leg curl, shoulder press, biceps curl, triceps bench press, abdominal crunch	3 × 10 reps of each exercise, 90 s rest between sets	100% of 10-RM	Rest & 40 and 60 min post; Doppler probes, ECG, BioPac MP100	**cf-PWV** No rest-post differences Response different compared to AER	10
Fahs et al., 2009	Healthy young men (*n =* 17)	Bench press, biceps curl	10 reps of bench press warm-up **Bench press** 4 × 5 reps **Biceps curl** 4 × 10 reps 2 min rest between sets	50% of 1-RM for warm-up **Bench press** 80% of 1-RM **Biceps curl**: 70% of 1-RM	Rest & within 15 min post; SphygmoCor/AT	**cf-PWV, AIx and AIx75** Greater within 15 min post	12
Figueroa and Vicil, 2011	Healthy young men (*n =* 11) and women (*n =* 12)	Bilateral leg extension, leg curl without vascular occlusion	3 × 10 reps of each bilateral leg extension and leg curl	40% of 1-RM	Rest & 0–2 min and 30 min post; SphygmoCor/AT	**AIx** Lower at 30 min post No difference between conditions	9
Heffernan et al., 2006	Healthy young men (*n =* 7) and women (*n =* 6)	Unilateral leg press (dominant limb)	6 sets to volitional fatigue	85% of 1-RM	Rest & 5 and 25 min post; SphygmoCor/AT	**cf-PWV** No rest-post differences	8
Heffernan et al., 2007d	Healthy young men (*n =* 13)	Bench press, bent-over row, leg extension, leg curl, shoulder press, biceps curl, triceps bench press, abdominal crunch	3 × 10 reps of each exercise, 90 s rest between sets	100% of 10-RM	Rest & 20 min post; Doppler probes, ECG, BioPac MP100	**cf-PWV** Greater at 20 min post Response different compared to AER	9
Heffernan et al., 2007b	Healthy young men (*n =* 14)	Unilateral leg press and leg extension	15 × 10 reps with alternating legs 70 s rest between sets	75% of 1-RM	Rest & 20 min post; SphygmoCor/AT	**cf-PWV** No rest-post differences	9
Kingsley et al., 2016	Healthy young men (*n =* 11) and women (*n =* 5)	Squat, bench press, and deadlift	3 × 10 reps of each exercise 2 min rest between sets	75% of 1-RM,	Rest & 10 min post; SphygmoCor/AT	**cf-PWV** Greater at 10 min post	10
Lydakis et al., 2008	Healthy young men (*n =* 7) and women (*n =* 8)	Unilateral knee extension	To volitional fatigue	Resistance increase by 10 W (men) and 5W (women) every 2 min	Rest & 0 min post; SphygmoCor/AT	**AIx** No rest-post differences	7
Mak and Lai, 2015	Healthy young men (*n =* 18)	Unilateral biceps curl without VM	10 × 10 reps 90 s between sets	75% of 1-RM	Rest & 0 and 15 min post; Esaote MyLabSat Ultrasound system	**cf-PWV** Greater at 0 min post with VM	8
Tai et al., 2018	Healthy young men (*n =* 12) and women (*n =* 3)	Squat, bench press and deadlift	3 × 10 reps of each exercise 2 min rest between sets	75% of 1-RM,	Rest & 10–20 min post; SphygmoCor/AT	**AIx, AIx75** Greater at 10–20 min post	12
Thiebaud et al., 2016	Healthy young men (YG, *n =* 12)	Leg press, chest press, knee flexion, lat pull down, knee extension	3 × 10 reps 2–3 min rest between sets and 2 min rest between exercises	65% of 1-RM	Rest & 5 min post; SphygmoCor/AT	**cf-PWV** No rest-post differences **AIx75** No rest-post differences	11
Yoon et al., 2010	Healthy young men (*n =* 13)	Bench press, squat, lat pull down, biceps curl, leg extension, leg curl, upright row, triceps extension	2 × 15 reps	60% of 1-RM	Rest & 20 and 40 min post; SphygmoCor/AT	**cf-PWV, AIx75** Greater at 20 min post **AIx** No rest-post differences	9

*Data are presented as mean ± SD. Reps, repetitions; AT, applanation tonometry; cf-PWV, carotid-femoral pulse wave velocity; P1, protocol 1; P2, protocol 2; ECG, electrocardiogram; AIx, augmentation index; AER, aerobic exercise; VM, Valsalva maneuvre; YG, young group*.

* *Differences reported at a statistically significant level p ≤ 0.05*.

**Table 4:** Summary of studies that examined carotid-femoral pulse wave velocity and/or augmentation indices post control (seated rest) intervention.

**Study**	**Participants**	**Intervention**	**Duration**	**Assessment time points; Method used**	Results (Group/time differences)^*^	**Quality score**
Barnes et al., 2010	Heathy young men (*n =* 11)	Quiet, seated rest	25 min	Rest & 90 min, 24, 48 and 72 h post; Colin VP2000/AT	**cf-PWV** No rest-post differences	8
Figueroa and Vicil, 2011	Healthy young men (*n =* 11) and women (*n =* 12)	Seated rest	not reported	Rest & 0–2 min and 30 min post; SphygmoCor/AT	**AIx** No rest-post differences	9
Kingsley et al., 2016	Healthy young men (*n =* 11) and women (*n =* 5)	Supine rest	30 min	Rest & 10 min post; SphygmoCor/AT	**cf-PWV** No rest-post differences	10
Kingwell et al., 1997	Healthy young men (*n =* 12)	Armchair reading	30 min	Rest & 30 and 60 min post; Custom-built software/AT	**cf-PWV** No rest-post differences	9
Lin et al., 2016	Healthy young men (*n =* 11)	Seated rest	Not reported	Rest & 90 min, 24, 48 and 72 h post; Millar Inc-Biopac/AT	**cf-PWV** No rest-post differences	10
Tai et al., 2018	Healthy young men (*n =* 12) and women (*n =* 3)	Supine rest	30 min	Rest & 10–20 min post; SphygmoCor/AT	**AIx, AIx75** No rest-post differences	12
Thiebaud et al., 2016	Healthy young men (YG, *n =* 12)	Seated rest	~ 20 min (+20 min waiting period)	Rest & 10 min post waiting period; SphygmoCor/AT	**cf-PWV, AIx75** No rest-post differences	11
Yoon et al., 2010	Healthy young men (*n =* 13)	Seated rest	Not reported	Rest & 20 and 40 min post; SphygmoCor/AT	**cf-PWV, AIx, AIx75** No rest-post differences	9

*Data are presented as mean ± SD. AT, applanation tonometry; cf-PWV, carotid-femoral pulse wave velocity; AIx, augmentation index; AIx75, augmentation index corrected for heart rate; YG, young group*.

* *Differences reported at a statistically significant level p ≤ 0.05*.

Exercise intensity was expressed in relative (i.e., a percentage of maximum heart rate) or absolute (i.e., Watt or km h^−1^) terms, and duration was either limited by time (10–60 min) or participant fatigue. Resistance exercise was executed using lower body only (four studies), upper body only (two studies), or whole-body exercise (seven studies) with varying repetitions (5 to fatigue), sets (1–15), intensities (10–110% of one repetition maximum) and rest periods (70 s to 4 min).

Outcome measures were assessed as follows: cf-PWV only in 23 studies (18 aerobic, 7 resistance, 2 assessed both exercise modes), AIx only in 4 studies (2 aerobic, 2 resistance), AIx75 only in 2 studies (2 aerobic), cf-PWV, and AIx/AIx75 in 9 studies (8 aerobic, 1 resistance), AIx and AIx75 in 3 studies (2 aerobic, 1 resistance), and cf-PWV, AIx, and AIx75 in 4 studies (2 aerobic, 2 resistance). In summary, 29 studies (64.4%) reported only one, 12 studies (26.7%) reported two and 4 studies (8.9%) reported three outcome measures.

The timing of outcome measures varied amongst studies from 0 min to 72 h; however, most (93.3%) examined variables at 0–30 min (91.2% of aerobic, 84.6% of resistance, 75% of the control group) of the post-intervention period. Time points for studies included in the meta-analysis varied from 0 to 60 min for aerobic studies and from 0 to 40 min for resistance studies. There was a weak, but significant, negative correlation (*r* = −0.368, *n* = 37, *p* = 0.02) between timing of post-intervention measurement and pre-post change (%) for cf-PWV following aerobic exercise only, indicating that early post-intervention measurements were more likely to demonstrate increased cf-PWV compared to a later measurement. No significant correlations were found for other outcome measures and following resistance exercise.

### Qualitative analysis

Mean score for methodological quality was 8.6 ± 1.4 (range 5–12) out of a possible maximum score of 13. The main reasons for poor scoring were: (a) lack of randomization of participants to interventions (26 studies, 57.8%) largely due to study design (i.e., pre-post intervention studies with only one intervention; (b) lack of control group (37 studies, 82.2%); and (c) no reporting of dropout/participation rates (24 studies, 53.3%). All but five studies (Kingwell et al., 1997; Munir et al., 2008; Figueroa and Vicil, 2011; Sun et al., 2015; Siasos et al., 2016a) discussed limitations and sources of potential bias. Risk of bias assessment via the Cochrane Collaboration's tool indicated that most information was from studies exhibiting overall low or unclear risk of bias (Supplementary Table 1).

### Meta-analyses of pre-post intervention change

#### Carotid femoral pulse wave velocity

**Figure 2:**
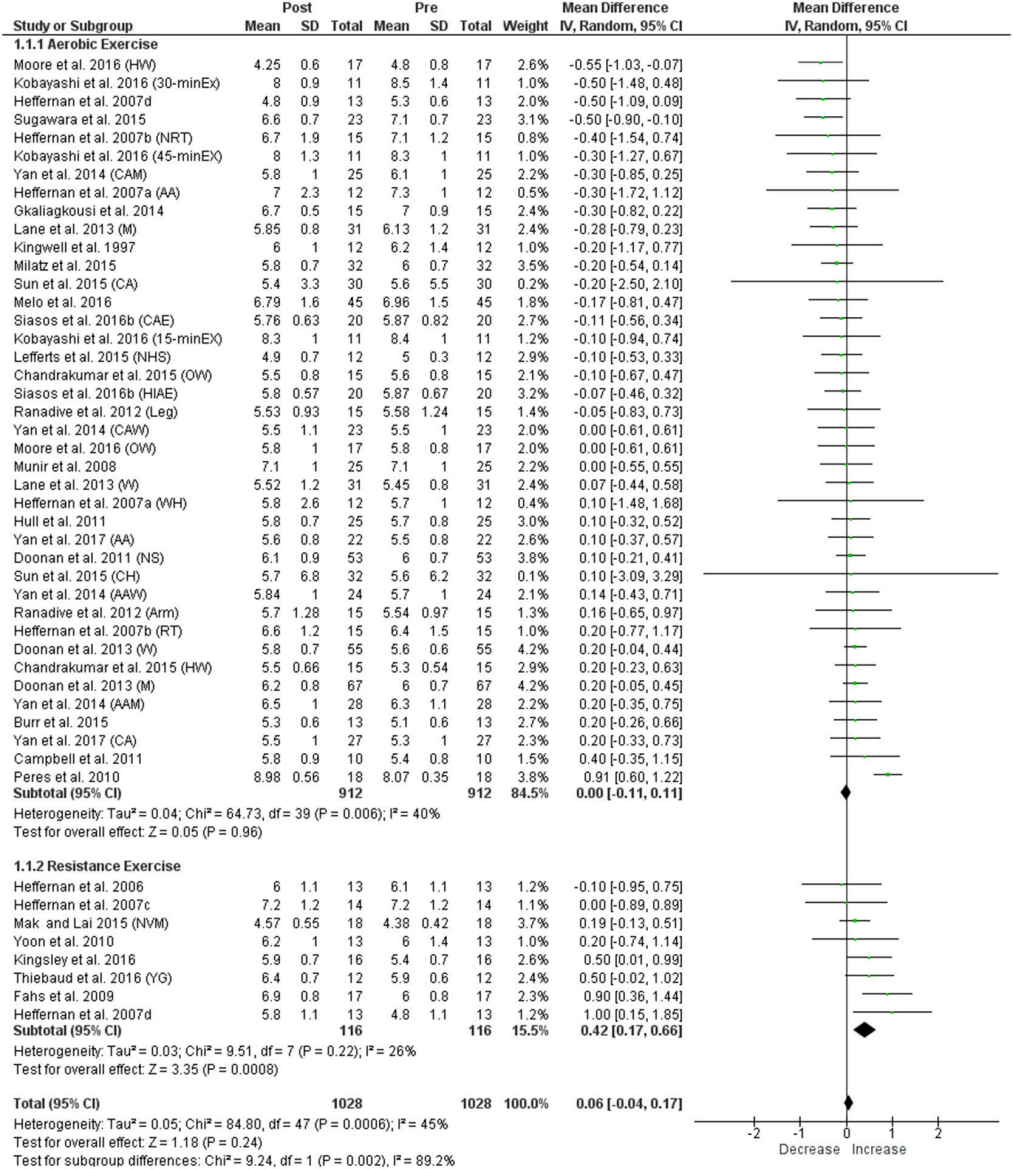
Forest plots showing the effect of acute aerobic and resistance exercise on cf-PWV. HW, healthy weight; 30-minEX, 30-min exercise duration; NRT, non-resistance trained; 45-minEX, 45-min exercise duration; AA, African American; CAM, Caucasian men; M, men; CA, Caucasian; CAE, continuous aerobic exercise; 15-minEX, 15-min exercise duration; NHS, no heat stress; HIAE, high-intensity aerobic exercise; Leg, leg ergometry; OW, overweight; CAW, Caucasian women; W, women; WH, white; NS, non-smokers; CH, Chinese; AAW, African American women; Arm, arm ergometry; RT, resistance trained; AAM, African-American men; NVM, no Valsalva maneuvre; YG, young group.

**Figure 3:**
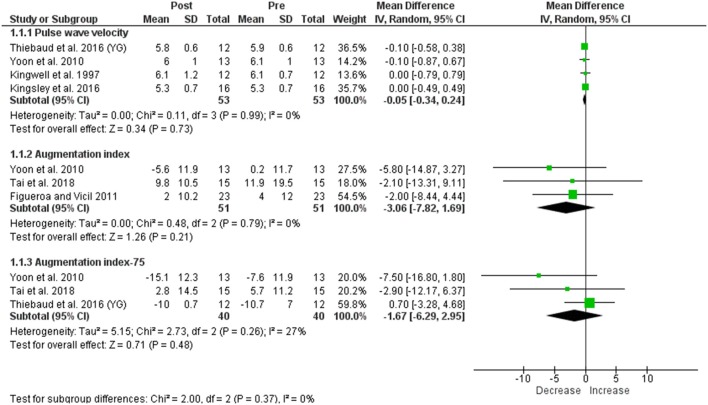
Forest plots showing the effect of seated rest on cf-PWV, AIx, and AIx75. YG, young group.

No significant change in cf-PWV was identified following aerobic exercise, and we found significant heterogeneity within this comparison (Figure 2). In contrast, resistance exercise was associated with significantly greater cf-PWV and non-significant heterogeneity. The pooled data for cf-PWV following aerobic and resistance exercise demonstrated an overall non-significant effect with significant heterogeneity among the studies overall (Figure 2). Heterogeneity between the two sub-groups (aerobic and resistance) was also significant (Figure 2). For the control group, no significant change in cf-PWV was observed, and heterogeneity between the studies was non-significant (Figure 3). Visual inspection of the funnel plot (Supplementary Figure 2A) did not suggest publication bias for resistance exercise but showed asymmetry on the right for aerobic exercise, suggesting the absence of studies with positive/increased cf-PWV, either because of publication bias or because of a true nonexistence of these studies (i.e., absence of publication bias).

#### Pulse wave reflection

**Figure 4:**
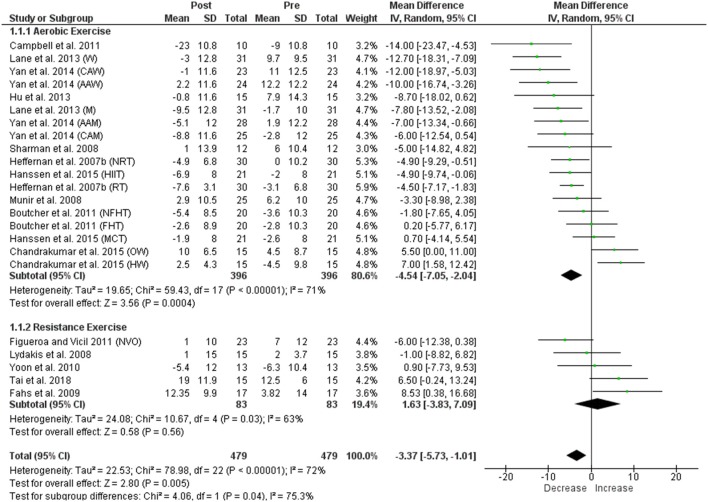
Forest plots showing the effect of acute aerobic and resistance exercise on AIx. W, women; CAW, Caucasian women; AAW, African American women; M, men; AAM, African American men; CAM, Caucasian men; NRT, non-resistance trained; HIIT, high-intensity interval training; RT- resistance trained; NFHT, no family history of hypertension; FHT, family history of hypertension; MCT, moderate continuous training; OW, overweight; HW, healthy weight; NVO, no vascular occlusion.

A significant reduction in AIx was identified following aerobic exercise, and we found significant heterogeneity within this comparison (Figure 4). In contrast, resistance exercise was not associated with a significant change in AIx, but significant heterogeneity among the studies existed (Figure 4). The pooled data for AIx following aerobic and resistance exercise demonstrated an overall significant reduction with significant heterogeneity among the studies overall (Figure 4). Heterogeneity between the two sub-groups (aerobic and resistance) was significant (Figure 4). For the control group, no significant change in AIx was observed, and heterogeneity between the studies was non-significant (Figure 3). Visual inspection of the funnel plot (Supplementary Figure 2B) did not suggest publication bias for aerobic exercise but showed asymmetry on the left for resistance exercise, suggesting the absence of studies with negative/decreased AIx, either because of publication bias or because of a true nonexistence of these studies (i.e., absence of publication bias). Since decreased AIx would be a desirable outcome following resistance exercise, it is unlikely that studies finding such reduction would remain unpublished; the asymmetry may therefore, reflect a true nonexistence.

**Figure 5:**
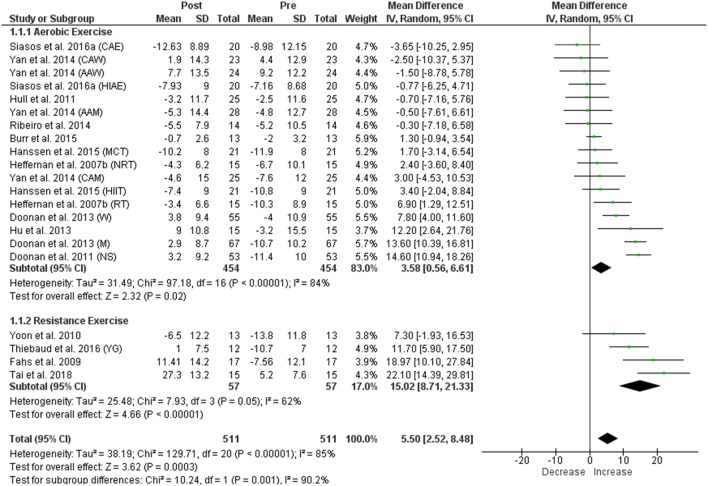
Forest plots showing the effect of acute aerobic and resistance exercise on AIx75. CAE, continuous aerobic exercise; CAW, Caucasian women; AAW, African American women; HIAE, high-intensity aerobic exercise; AAM, African American men; MCT, moderate continuous training; NRT, non-resistance trained; CAM, Caucasian men; HIIT, high-intensity interval training; RT, resistance trained; W, women; M, men; NS, non-smokers; YG, young group.

A significant increase in AIx75 was identified following both aerobic and resistance exercise, and we found significant heterogeneity within each comparison (Figure 5). The pooled data for AIx75 following aerobic and resistance exercise demonstrated an overall significant increase with significant heterogeneity among the studies overall (Figure 5). Heterogeneity between the subgroups (aerobic and resistance) was significant (Figure 5). For the control group, no significant change in AIx75 was observed, and heterogeneity between the studies was non-significant (Figure 3). Visual inspection of the funnel plot (Supplementary Figure 2C) showed asymmetry on the right for aerobic exercise, suggesting the absence of studies with positive/increased AIx75, either because of publication bias or because of a true non-existence of these studies (i.e., absence of publication bias). For resistance exercise, the funnel plot showed asymmetry on the left for resistance studies, suggesting the absence of studies with negative/decreased AIx75, either because of publication bias or because of a true nonexistence of these studies (i.e., absence of publication bias). Since decreased AIx75 would be a desirable outcome following resistance exercise, it is unlikely that studies finding such a reduction would remain unpublished; the asymmetry may therefore, reflect a true nonexistence.

## Discussion

The current systematic review and meta-analysis demonstrated that, overall, distinct arterial stiffness recovery responses existed following a single bout of aerobic and resistance exercise with the differences possibly originating from unique cardiovascular (i.e., blood pressure, heart rate) and non-cardiovascular (i.e., inflammatory products) processes. Additionally, the results from the present review and meta-analysis demonstrate the limitations of current research designs that can assist in improving the experimental approach of future studies.

### Carotid-femoral pulse wave velocity

Unlike the mean difference following resistance exercise, mean difference for cf-PWV following aerobic exercise in the present meta-analysis was not significant overall, which suggested an inability for aerobic exercise to significantly alter arterial stiffness responses. Although the exact mechanisms regulating modal differences in cf-PWV have not been identified, several mechanisms have been implicated in previous studies (Yoon et al., 2010) and likely involve distinct blood pressure responses to exercise (Izzo, 2004).

While blood pressure increases during both acute aerobic and resistance exercise, the magnitude and nature of this increase differs between modes (MacDougall et al., 1985). Increases in blood pressure during aerobic exercise can be moderate and more sustained due to the use of large muscles in a rhythmic manner whereas intense resistance exercise can result in brief, intermittent increases in blood pressure, reaching up to fourfold resting values, due to mechanical compression of blood vessels, a strong exercise pressor reflex, and execution of the Valsalva maneuver (MacDougall et al., 1985). Subsequently, this extreme, intermittent distending pressure may cause a transient switch in load bearing from elastin to collagen fibers of arteries and thus, increased cf-PWV (MacDougall et al., 1985), which is not seen with the moderate and stable pressure during aerobic exercise. However, the significant increase in cf-PWV was not observed in all resistance exercise studies. Studies that employed upper and whole-body resistance exercise reported increased cf-PWV (Heffernan et al., 2007d; Fahs et al., 2009; Kingsley et al., 2016), whereas studies investigating lower body resistance exercise reported no change (Heffernan et al., 2006, 2007c). These findings were possibly facilitated by the greater blood pressure changes during upper body compared to lower body resistance exercise (Stenberg et al., 1967), highlighting the influence of muscle groups exercised rather than intensity (Heffernan et al., 2007c) as a key contributor to changes in cf-PWV. The addition of the Valsalva maneuver, almost obligatory during intense (>80–85% of one repetition maximum) resistance exercise (McCartney, 1999), may also contribute independently to the increase in cf-PWV through promotion of large increases in intra-thoracic and intra-abdominal pressures, which are transmitted to the aorta (MacDougall et al., 1985; Heffernan et al., 2007c).

Reduced left ventricular ejection time, as seen with tachycardia during and following acute exercise, has also been implicated as an independent predictor of cf-PWV with Salvi et al. (2013) reporting a significant and inverse association between cf-PWV and left ventricular ejection time for all age groups. Previous studies indicated that heart rate values during intense aerobic exercise remained below these reached during resistance exercise with heart rates of 147 beats per minute reported during high-intensity cycling (80% of maximum heart rate) (Sharman et al., 2005) compared to 160–170 beats per minute reported during each set of a lift (McCartney, 1999)_._ One suggested mechanism for the left ventricular ejection time and cf-PWV association was the greater left ventricular, ejection force during a reduced left ventricular ejection time with this increased force translating to augmented blood pressure and increased cf-PWV as a result of changes in arterial wall viscoelastic properties as described above. Additionally, reduced arterial recoil time for predominantly elastic arteries (e.g., aorta) resulting from tachycardia may also contribute to vascular stiffening (Mangoni et al., 1996). Together, changes in blood pressure, heart rate, and left ventricular ejection time may be crucial cardiovascular modulators of cf-PWV following different modes of acute exercise; however, non-cardiovascular factors may also modify cf-PWV post-exercise.

Compared to more concentrically-biased aerobic activities (e.g., cycling), substantially elevated inflammation (Barnes et al., 2010) was reported following the greater eccentric component of acute resistance exercise that may also be partially responsible for increased cf-PWV following resistance exercise. Specifically, elevated levels of acute inflammatory markers such as c-reactive protein, interleukin-6, and matrix metalloproteinase-9 were associated with significantly increased cf-PWV (Yasmin et al., 2004; Vlachopoulos et al., 2005; Shirwany and Zou, 2010; Jae et al., 2013). Potentially, eccentric muscle contractions during resistance exercise and eccentrically-biased aerobic exercise protocols such as downhill treadmill running (Burr et al., 2015) may induce greater muscle damage and inflammatory products that negatively influence nitric oxide bioavailability (Vlachopoulos et al., 2005) and endothelium-dependent dilatation (Hingorani et al., 2000), thereby enhancing arterial stiffness (Kano et al., 2005). Further, resistance exercise has been reported to promote the release of angiotensin II (Kraemer et al., 1999). Besides its vasoconstrictive effect, angiotensin II may cause a shift from elastin to collagen synthesis, vascular hypertrophy, a heightened inflammatory response, and depression of nitric oxide dependent signaling (Zieman et al., 2005). This alteration in endothelial cell signaling and vascular smooth muscle tone has been demonstrated to affect vascular stiffening and, particularly, cf-PWV (Rehman et al., 2002). In addition to these reported cardiovascular and non-cardiovascular mechanisms modulating cf-PWV, methodological factors such as timing of post-exercise measurement, and duration and intensity of exercise may also explain the absence of significant changes in cf-PWV following aerobic exercise.

Previously, increased cf-PWV was reported for both healthy young men and women at 2-min following a treadmill protocol to volitional exhaustion (Doonan et al., 2013), whereas other studies (Gkaliagkousi et al., 2014; Melo et al., 2016) reported no change in cf-PWV at 10, 30, and 60 min following aerobic exercise. Exercise protocols and study participants were nearly identical amongst these studies with the divergent findings possibly resulting from the timing of measurement. Our finding of a weak, but significant, negative correlation between timing of post-intervention measurement and cf-PWV outcomes following aerobic exercise indicates that early post-intervention measurements were more likely to demonstrate increased cf-PWV compared to later measurements. A recent systematic review of acute aerobic exercise studies also reported that timing of post-exercise measurement possibly influenced arterial stiffness outcomes, since changes may be only short-lived (Mutter et al., 2016). Therefore, measurement of arterial stiffness indices early during post-intervention may allow the detection of significant changes that may have abated later during post-intervention (e.g., 10-min). No significant correlation between timing of post-intervention measurement and cf-PWV following resistance exercise was evident in this review, indicating a more consistent effect of acute resistance exercise on cf-PWV, most likely due to the severity of cardiovascular disturbance during exercise (MacDougall et al., 1985; McCartney, 1999; Sharman et al., 2005). Therefore, the characteristics of the exercise bout were likely to be crucial to the post-exercise response. In the current review, exercise durations varied between studies (10–60 min) with one aerobic exercise study (Kobayashi et al., 2017) reporting a prolonged reduction in cf-PWV following a 60-min protocol compared to a 15- and 30-min protocol. However, many studies failed to report exercise duration which limited conclusions about the effect of exercise duration on post-exercise change in cf-PWV. Additionally, exercise intensity may be a significant factor that contributes to changes in post-exercise cf-PWV. Similar to duration, intensity of exercise bouts varied between studies with the nature of some aerobic interventions (i.e., graded exercise protocols) and the diversity of exercise intensity measures used limiting a meaningful comparison within and between exercise modes in the current review. Future studies, including systematic reviews and meta-analyses, are encouraged to report and consider both duration and intensity of the exercise bout when examining post-exercise cf-PWV.

While attention to the exercise bout itself should be considered, factors such as diversity of measuring techniques and participants may also be crucial to the current cf-PWV findings. Different assessment techniques, including automated devices, applanation tonometry/calculation, and direct/subtraction methods, have been utilized with variable findings after aerobic exercise. Further, several studies (Lydakis et al., 2008; Munir et al., 2008; Peres et al., 2010; Hull et al., 2011; Ranadive et al., 2012; Hu et al., 2013; Gkaliagkousi et al., 2014; Burr et al., 2015; Sun et al., 2015; Melo et al., 2016; Kingsley et al., 2017; Yan et al., 2017; Tai et al., 2018) failed to report separate cf-PWV values for female and male participants with the known inherent sex differences in cf-PWV at rest and following exercise (Doonan et al., 2013) potentially impeding valid comparisons between studies.

In summary, acute resistance exercise induces adverse effects on cf-PWV due to cardiovascular and/or non-cardiovascular mechanisms. In contrast, acute aerobic exercise effects were minimal due to these cardiovascular and/or non-cardiovascular mechanisms with findings also potentially influenced by exercise protocols (e.g., muscle groups exercised), timing of measurement, duration and intensity of exercise, measuring techniques, and/or participants that remain to be elucidated.

### Wave reflection indices

Overall, the mean difference for AIx was large and negative following aerobic exercise, representing a substantial reduction in wave reflection, whereas it was small following resistance exercise, indicating no acute change. Results from studies investigating acute aerobic exercise were largely homogenous with most studies reporting decreased AIx. Previously, aerobic exercise was reported to promote nitric oxide-induced vasodilation via increased blood flow and shear stress, resulting in reduced wave reflection (Munir et al., 2008). The current meta-analysis provides further support of this beneficial effect of aerobic exercise on wave reflection. In contrast, AIx results from resistance exercise studies were inconsistent, with results possibly a result of distinct muscle group differences. Whereas upper and whole body resistance exercise were associated with increased wave reflection (Fahs et al., 2009; Tai et al., 2018), lower body resistance exercise was associated with reduced wave reflection (Figueroa and Vicil, 2011), implying that muscle group activation and/or changes in cardiovascular and non-cardiovascular function resolved the effect of acute resistance exercise on indices of wave reflection. Previous measures of wave reflection were determined by the velocity of the incident wave (i.e., cf-PWV), left ventricular ejection time, and peripheral vasomotor tone (Kelly et al., 2001; London and Pannier, 2010). Although the greater systolic and, consequently, shorter diastolic duration associated with increased left ventricular ejection time also increases the probability of an early wave return (London and Pannier, 2010), cf-PWV may be the predominant influence on wave reflection timing (Kelly et al., 2001). Additionally, as magnitude of wave reflection is affected by a mismatch between central and peripheral vasomotor tone, the greater peripheral vessel constriction seen with resistance exercise may result in greater wave reflection (Kelly et al., 2001). Greater activation of the sympathetic nervous system during resistance exercise, and specifically during upper body resistance exercise, has been identified as a key mechanism for the vasoconstrictive effect (Okamoto et al., 2009). Therefore, the greater wave reflection and increased AIx reported with upper and whole-body resistance exercise may result from increased cf-PWV and/or greater peripheral vasomotor tone.

The current review extensively evaluated the acute influence of exercise mode on indices of arterial stiffness and wave reflection, including AIx75, which has rarely been examined in terms of exercise mode. Based upon the meta-analysis, AIx75 was overall significantly increased following both acute aerobic and resistance exercise with the mean difference following resistance exercise nearly five times that following aerobic exercise (15.02 vs. 3.54%). This considerably greater and non-heart rate mediated (i.e., cf-PWV, left ventricular ejection time, and peripheral vasomotor tone) response following resistance exercise further exemplified the modal differences in arterial stiffness and wave reflection responses. Additionally, these results indicated that AIx75 may be a more useful measure of left ventricular afterload, compared to AIx, when comparing exercise responses in future studies.

### Methodological considerations and recommendations for future studies

The meta-analysis reported here combined data from many studies to estimate acute intervention effects on arterial stiffness and wave reflection with more precision than possible in a single study. The main limitation of this approach was the heterogeneity amongst studies in terms of participants, exercise protocols (i.e., exercise mode, duration and intensity), and outcome assessment (e.g., timing of measurement). The modified PEDro scale and risk of bias assessment using the Cochrane tool identified several possible sources for risk of bias within the studies. For example, lack of random sequence generation in 57.8% of all studies (item 2 on PEDro scale), largely due to study design (i.e., pre-post intervention design without control intervention) and lack of allocation concealment (item 3), may have caused an unclear risk of selection bias. Similarly, the nature of the interventions, that is aerobic, resistance or no exercise, made blinding of participants and outcome assessors impossible, which may have led to performance or detection bias. However, participants were likely unaware of the expected outcome associated with their individual intervention or the mechanisms affecting these outcomes; therefore, attempts at manipulating outcomes were unlikely. Assessors may have been aware of expected outcomes with each intervention, but automated measurement techniques of cf-PWV and wave reflection likely minimized the possibility of detection bias. A lack of reporting of dropout/participation rates (item 12) in more than 50% of studies raised the issue of attrition bias. However, no trend between any components of bias and mean difference was identified.

During this review, several common, methodological concerns were also noted. Firstly, very few studies employed a specific exercise protocol recommended for improving health of the general population (Garber et al., 2011). This selection of exercise protocols may greatly restrict the applicability and generalizability of findings from prior studies with future studies encouraged to examine exercise modalities and intensities/durations prescribed for general and cardiovascular health. Secondly, more than 50% of studies were designed as non-randomized, pre-post interventions without a non-exercise control group, thereby weakening internal validity (Harris et al., 2006). A mere 17.7% (8/45) of studies included a control group to account for normal variation in arterial stiffness. A randomized, cross-over design including a control group may be a more appropriate study design to avoid potential threats for establishing causality in future studies (Harris et al., 2006). Thirdly, measurements of post-exercise arterial stiffness in most studies were conducted at isolated time points rather than over a time course. As changes in post-exercise arterial stiffness and wave reflection indices are generally transient in nature (Mutter et al., 2016), prior studies may have “missed” these real changes and reported erroneous conclusions. Future studies could avoid this shortcoming by adopting a schedule that incorporates measurements at regular intervals over a time course (e.g., every 10-min). Fourth, measurement of only one index was conducted in most studies and may have not provided a comprehensive picture of changes in cardiovascular loading, particularly as AIx has been implicated as a more sensitive marker in younger adults, while cf-PWV is considered a more meaningful marker in older adults (Mitchell et al., 2004). Finally, several studies did not report results separately for female and male participants. Despite some efforts to control for menstrual cycle phase (Madhura and Sandhya, 2014), the apparent difference in resting cf-PWV and cf-PWV responses to exercise between males and females (Doonan et al., 2013) may have impacted on arterial stiffness and wave reflection results. More importantly though, most of the included studies did not look at exercise modal differences in the same population within the same study. Therefore, the observed exercise modal differences may be due to confounding factors such as different participant characteristics.

### Limitations

Like most reviews, several limitations need to be acknowledged. The current systematic review and meta-analysis was based upon studies reported in English. The use of English search terms may have led to omission of studies reported in other languages. To minimize the risk of English-language bias, several databases, including The Cochrane Controlled Trials Register that has been reported as the best single source of trials for inclusion in systematic reviews and meta-analyses (Egger et al., 1997), were searched. Additionally, reference lists were searched manually to identify potential further trials, which has been described as potentially more important in finding further trials than the choice of electronic database (Egger et al., 1997). Furthermore, due to great heterogeneity in post-exercise measurement time points, our meta-analysis was based on data within a post-intervention period rather than at one time point. Of the 45 included studies, 41 reported at least one measurement time point within a 60-min post-intervention period with no consistent time point identified for most studies. Consequently, the last reported measurement within the 60-min period was used in the calculation of pre-post mean differences for consistency of comparisons in post-exercise assessment of arterial stiffness indices.

### Clinical implications

Increased central arterial stiffness (i.e., cf-PWV) and wave reflection measures (i.e., AIx/AIx75) following acute resistance exercise were observed for healthy adults in the present meta-analysis. This transient increase may not be cause for concern in a young, healthy population group with low baseline levels of central arterial stiffness, as the observed, average 0.46 ms^−1^ increase in cf-PWV was well below the 1 ms^−1^ increase associated with a 15% increase in CVD risk (Vlachopoulos et al., 2010). Nevertheless, these arterial stiffness and wave reflection alterations may contribute transiently to an increased risk of cardiovascular events seen with acute, strenuous exercise in older, high-risk populations (Willich et al., 1993; Hallqvist et al., 2000). Despite the well-known benefits of resistance exercise on muscular strength and endurance, functional capacity and quality of life (Garber et al., 2011), the safety of resistance exercise on arterial stiffness indices and subsequent cardiovascular risk for individuals with unstable medical conditions has yet to be clearly established. Similar to aerobic exercise, cardiovascular risks associated with resistance exercise are likely determined by an individual's physical fitness and activity level, age, exercise intensity, and existing cardiovascular conditions (Williams et al., 2007). Specifically, resistance exercise is contraindicated in individuals with uncontrolled or high-risk, pre-existing cardiovascular conditions, highlighting the importance of vigilant patient screening and monitoring of strenuous exercise in at-risk populations prior to and during exercise (Williams et al., 2007). Future studies will elucidate the effect of acute resistance exercise on arterial stiffness and wave reflections in individuals with cardiovascular risk factors and CVD for improved cardiovascular health.

## Conclusions

In conclusion, distinct arterial stiffness recovery responses were identified following a single acute bout of aerobic and resistance exercise. Overall, acute aerobic exercise did not change cf-PWV but resulted in reduced AIx and increased AIx75. In contrast, acute resistance was likely to induce an adversarial effect on arterial stiffness with overall increases in both cf-PWV and AIx75, potentially arising from cardiovascular and non-cardiovascular factors. Common limitations of current research designs, including great diversity in exercise protocols, selective timing of measurements, and lack of control group should be addressed in future studies to facilitate interpretation and improve generalizability of arterial stiffness findings to cardiovascular health.

## Author contributions

All authors contributed ideas to the design of this study and developed the search strategy. DP conducted the literature search and the study quality assessment. All authors decided on the final selection of studies to be included in this review and meta-analysis. DP extracted study information and outcome data. KD performed the statistical analyses. DP developed the first paper draft, and all authors revised the manuscript for important intellectual content and approved the final version of the article.

### Conflict of interest statement

The authors declare that the research was conducted in the absence of any commercial or financial relationships that could be construed as a potential conflict of interest.

## Supplementary material

Supplementary Figure 1 The PubMed search strategy employed in the current review.

Supplementary Figure 2 Funnel plot analyses for studies investigating cf-PWV **(A)**. AIx **(B)** and AIx75 **(C)**.

Supplementary Table 1 Risk of Bias summary.

## Abbreviations

AIx: Augmentation index
AIx75: Augmentation index corrected for heart rate of 75 beats per minute
cf-PWV: Carotid-femoral pulse wave velocity
CVD: Cardiovascular disease
SD: Standard deviation.

## References

[B1] Ashor A. W. Lara J. Siervo M. Celis-Morales C. Mathers J. C. (2014). Effects of exercise modalities on arterial stiffness and wave reflection: a systematic review and meta-analysis of randomized controlled trials. PLoS ONE 9:e11034. 10.1371/journal.pone.0110034 25333969 PMC4198209

[B2] Barnes J. N. Trombold J. R. Dhindsa M. Lin H.-F. Tanaka H. (2010). Arterial stiffening following eccentric exercise-induced muscle damage. J. Appl. Physiol. 109, 1102–1108. 10.1152/japplphysiol.00548.2010 20671032

[B3] Boutcher Y. N. Hopp J. P. Boutcher S. H. (2011). Acute effect of a single bout of aerobic exercise on vascular and baroreflex function of young males with a family history of hypertension. J. Hum. Hypertens. 25, 311–319. 10.1038/jhh.2010.62 20555357

[B4] Burr J. F. Boulter M. Beck K. (2015). Arterial stiffness results from eccentrically biased downhill running exercise. J. Sci. Med. Sport 18, 230–235. 10.1016/j.jsams.2014.03.003 24709362

[B5] Campbell R. Fisher J. P. Sharman J. E. McDonnell B. J. Frenneaux M. P. (2011). Contribution of nitric oxide to the blood pressure and arterial responses to exercise in humans. J. Hum. Hypertens. 25, 262–270. 10.1038/jhh.2010.53 20505750

[B6] Cavalcante J. L. Lima J. A. Redheuil A. Al-Mallah M. H. (2011). Aortic stiffness: current understanding and future directions. J. Am. Coll. Cardiol. 57, 1511–1522. 10.1016/j.jacc.2010.12.017 21453829

[B7] Chandrakumar D. Boutcher S. H. Boutcher Y. N. (2015). Acute exercise effects on vascular and autonomic function in overweight men. J. Sports Med. Phys. Fitness 55, 91–102. 25642686

[B8] Collier S. R. Diggle M. D. Heffernan K. S. Kelly E. E. Tobin M. M. Fernhall B. (2010). Changes in arterial distensibility and flow-mediated dilation after acute resistance vs. aerobic exercise. J. Strength Cond. Res. 24, 2846–2852. 10.1519/JSC.0b013e3181e840e0 20885204

[B9] Davies J. I. Struthers A. D. (2003). Pulse wave analysis and pulse wave velocity: a critical review of their strengths and weaknesses. J. Hypertens. 21, 463–472. 10.1097/00004872-200303000-00004 12640232

[B10] Doonan R. J. Mutter A. Egiziano G. Gomez Y.-H. Daskalopoulou S. S. (2013). Differences in arterial stiffness at rest and after acute exercise between young men and women. Hypertens. Res. 36, 226–231. 10.1038/hr.2012.158 23051656

[B11] Doonan R. J. Scheffler P. Yu A. Egiziano G. Mutter A. Bacon S.. (2011). Altered arterial stiffness and subendocardial viability ratio in young healthy light smokers after acute exercise. PLoS ONE 6:e26151. 10.1371/journal.pone.0026151 22028821 PMC3189960

[B12] Egger M. Zellweger-Zähner T. Schneider M. Junker C. Lengeler C. Antes G. (1997). Language bias in randomised controlled trials published in English and German. Lancet 350, 326–329. 10.1016/S0140-6736(97)02419-7 9251637

[B13] Elkins M. R. Herbert R. D. Moseley A. M. Sherrington C. Maher C. (2010). Rating the quality of trials in systematic reviews of physical therapy interventions. Cardiopulm. Phys. Ther. J. 21, 20–26. 20957075 PMC2941354

[B14] Fahs C. A. Heffernan K. S. Fernhall B. (2009). Hemodynamic and vascular response to resistance exercise with L-arginine. Med. Sci. Sports Exerc. 41, 773–779. 10.1249/MSS.0b013e3181909d9d 19276857

[B15] Figueroa A. Vicil F. (2011). Post-exercise aortic hemodynamic responses to low-intensity resistance exercise with and without vascular occlusion. Scand. J. Med. Sci. Sports 21, 431–436. 10.1111/j.1600-0838.2009.01061.x 20136757

[B16] Franklin S. S. (2008). Beyond blood pressure: arterial stiffness as a new biomarker of cardiovascular disease. J. Am. Soc. Hypertens. 2, 140–151. 10.1016/j.jash.2007.09.002 20409896

[B17] Garber C. E. Blissmer B. Deschenes M. R. Franklin B. A. Lamonte M. J. Lee I. M.. (2011). Quantity and quality of exercise for developing and maintaining cardiorespiratory, musculoskeletal, and neuromotor fitness in apparently healthy adults: guidance for prescribing exercise. Med. Sci. Sports Exerc. 43, 1334–1359. 10.1249/MSS.0b013e318213fefb 21694556

[B18] Gkaliagkousi E. Gavriilaki E. Nikolaidou B. Triantafyllou G. Douma S. (2014). Exercise-induced pulse wave velocity changes in untreated patients with essential hypertension: the effect of an angiotensin receptor antagonist. J. Clin. Hypertens. 16, 482–487. 10.1111/jch.12340 24853292 PMC8031989

[B19] Hallqvist J. Möller J. Ahlbom A. Diderichsen F. Reuterwall C. de Faire U.. (2000). Does heavy physical exertion trigger myocardial infarction? A case-crossover analysis nested in a population-based case-referent study. Am. J. Epidemiol. 151, 459–467. 10.1093/oxfordjournals.aje.a010231 10707914

[B20] Hanssen H. Nussbaumer M. Moor C. Cordes M. Schindler C. Schmidt-Trucksäss A. (2015). Acute effects of interval versus continuous endurance training on pulse wave reflection in healthy young men. Atherosclerosis 238, 399–406. 10.1016/j.atherosclerosis.2014.12.038 25558034

[B21] Harris A. D. McGregor J. C. Perencevich E. N. Furuno J. P. Zhu J. Peterson D. E.. (2006). The use and interpretation of quasi-experimental studies in medical informatics. J. Am. Med. Inform. Assoc. 13, 16–23. 10.1197/jamia.M1749 16221933 PMC1380192

[B22] Heffernan K. S. Jae S. Y. Fernhall B. (2007a). Racial differences in arterial stiffness after exercise in young men. Am. J. Hypertens. 20, 840–845. 10.1016/j.amjhyper.2007.03.015 17679030

[B23] Heffernan K. S. Jae S. Y. Echols G. H. Lepine N. R. Fernhall B. (2007b). Arterial stiffness and wave reflection following exercise in resistance-trained men. Med. Sci. Sports Exerc. 39, 842–848. 10.1249/mss.0b013e318031b03c 17468584

[B24] Heffernan K. S. Jae S. Y. Edwards D. G. Kelly E. E. Fernhall B. (2007c). Arterial stiffness following repeated Valsalva maneuvers and resistance exercise in young men. Appl. Physiol. Nutr. Metab. 32, 257–264. 10.1139/h06-107 17486167

[B25] Heffernan K. S. Rossow L. Jae S. Y. Shokunbi H. G. Gibson E. M. Fernhall B. (2006). Effect of single-leg resistance exercise on regional arterial stiffness. Eur. J. Appl. Physiol. 98, 185–190. 10.1007/s00421-006-0259-9 16896730

[B26] Heffernan K. Collier S. Kelly E. Jae S. Fernhall B. (2007d). Arterial stiffness and baroreflex sensitivity following bouts of aerobic and resistance exercise. Int. J. Sports Med. 28, 197–203. 10.1055/s-2006-924290 17024636

[B27] Higgins J. Green S. (eds.). (2011). Cochrane Handbook for Systematic Reviews of Interventions Version 5.1.0. Available online at: http://handbook-5-1.cochrane.org/ (updated March, 2011).

[B28] Hingorani A. D. Cross J. Kharbanda R. K. Mullen M. J. Bhagat K. Taylor M.. (2000). Acute systemic inflammation impairs endothelium-dependent dilatation in humans. Circulation 102, 994–999. 10.1161/01.CIR.102.9.994 10961963

[B29] Hu M. Yan H. Ranadive S. M. Agiovlasitis S. Fahs C. A. Atiq M.. (2013). Arterial stiffness response to exercise in persons with and without Down syndrome. Res. Dev. Disabil. 34, 3139–3147. 10.1016/j.ridd.2013.06.041 23883823

[B30] Hull J. H. Ansley L. Bolton C. E. Sharman J. E. Knight R. K. Cockcroft J. R.. (2011). The effect of exercise on large artery haemodynamics in cystic fibrosis. J. Cystic Fibros. 10, 121–127. 10.1016/j.jcf.2010.12.001 21220217

[B31] Izzo J. J. L. (2004). Arterial stiffness and the systolic hypertension syndrome. Curr. Opin. Cardiol. 19, 341–352. 10.1097/01.hco.0000126581.89648.10 15218394

[B32] Jae S. Y. Yoon E. S. Jung S. J. Jung S. G. Park S. H. Kim B. S.. (2013). Effect of cardiorespiratory fitness on acute inflammation induced increases in arterial stiffness in older adults. Eur. J. Appl. Physiol. 113, 2159–2166. 10.1007/s00421-013-2648-1 23615823

[B33] Kadic A. J. Vucic K. Dosenovic S. Sapunar D. Puljak L. (2016). Extracting data from figures with software was faster, with higher interrater reliability than manual extraction. J. Clin. Epidemiol. 74, 119–123. 10.1016/j.jclinepi.2016.01.002 26780258

[B34] Kano Y. Padilla D. J. Behnke B. J. Hageman K. S. Musch T. I. Poole D. C. (2005). Effects of eccentric exercise on microcirculation and microvascular oxygen pressures in rat spinotrapezius muscle. J. Appl. Physiol. 99, 1516–1522. 10.1152/japplphysiol.00069.2005 15994245

[B35] Kelly R. P. Millasseau S. C. Ritter J. M. Chowienczyk P. J. (2001). Vasoactive drugs influence aortic augmentation index independently of pulse-wave velocity in healthy men. Hypertension 37, 1429–1433. 10.1161/01.HYP.37.6.1429 11408390

[B36] Kingsley J. D. P. D. Mayo X. M. S. Tai Y. L. M. S. Fennell C. M. S. (2016). Arterial stiffness and autonomic modulation following free-weight resistance exercises in resistance trained individuals. J. Strength Cond. Res. 30, 3373–3380. 10.1519/JSC.0000000000001461 27253837

[B37] Kingsley J. D. Tai Y. L. Vaughan J. A. Mayo X. (2017). High-intensity interval cycling exercise on wave reflection and pulse wave velocity. J. Strength Cond. Res. 31, 1313–1320. 10.1519/JSC.0000000000001598 27548787

[B38] Kingwell B. A. Berry K. L. Cameron J. D. Jennings G. L. Dart A. M. (1997). Arterial compliance increases after moderate-intensity cycling. Am. J. Physiol. 273(5 Pt 2), H2186–H2191. 10.1152/ajpheart.1997.273.5.H2186 9374752

[B39] Kobayashi R. Hatakeyama H. Hashimoto Y. Okamoto T. (2017). Acute effects of different aerobic exercise duration on pulse wave velocity in healthy young men. J. Sports Med. Phys. Fitness 57, 1695–1701. 10.23736/S0022-4707.16.06894-8 27849118

[B40] Kraemer W. J. Fleck S. J. Maresh C. M. Ratamess N. A. Gordon S. E. Goetz K. L.. (1999). Acute hormonal responses to a single bout of heavy resistance exercise in trained power lifters and untrained men. Can. J. Appl. Physiol. 24, 524–537. 10.1139/h99-034 10638340

[B41] Lane A. Ranadive S. Yan H. Kappus R. Cook M. Sun P.. (2013). Effect of sex on wasted left ventricular effort following maximal exercise. Int. J. Sports Med. 34, 770–776. 10.1055/s-0032-1329990 23526590

[B42] Lefferts W. K. Heffernan K. S. Hultquist E. M. Fehling P. C. Smith D. L. (2015). Vascular and central hemodynamic changes following exercise-induced heat stress. Vasc. Med. 20, 222–229. 10.1177/1358863X14566430 25939655

[B43] Li Y. Hanssen H. Cordes M. Rossmeissl A. Endes S. Schmidt-Trucksäss A. (2015). Aerobic, resistance and combined exercise training on arterial stiffness in normotensive and hypertensive adults: a review. Eur. J. Sport Sci. 15, 443–457. 10.1080/17461391.2014.955129 25251989

[B44] Liberati A. Altman D. G. Tetzlaff J. Mulrow C. Gøtzsche P. C. Ioannidis J. P. A.. (2009). The PRISMA statement for reporting systematic reviews and meta-analyses of studies that evaluate healthcare interventions: explanation and elaboration. BMJ 339:b2700. 10.1136/bmj.b2700 19622552 PMC2714672

[B45] Lin H. F. Tung K. Chou C. C. Lin C. C. Lin J. G. Tanaka H. (2016). Panax ginseng and salvia miltiorrhiza supplementation abolishes eccentric exercise-induced vascular stiffening: a double-blind randomized control trial. BMC Complement. Altern. Med. 16:168. 10.1186/s12906-016-1139-4 27266702 PMC4895816

[B46] London G. M. Pannier B. (2010). Arterial functions: how to interpret the complex physiology. Nephrol. Dial. Transplant. 25, 3815–3823. 10.1093/ndt/gfq614 20947536

[B47] Lydakis C. Momen A. Blaha C. Gugoff S. Gray K. Herr M.. (2008). Changes of central haemodynamic parameters during mental stress and acute bouts of static and dynamic exercise. J. Hum. Hypertens. 22, 320–328. 10.1038/jhh.2008.4 18273040

[B48] MacDougall J. D. Tuxen D. Sale D. G. Moroz J. R. Sutton J. R. (1985). Arterial blood pressure response to heavy resistance exercise. J. Appl. Physiol. 58, 785–790. 10.1152/jappl.1985.58.3.785 3980383

[B49] Madhura M. Sandhya T. (2014). Effect of different phases of menstrual cycle on reflection index, stiffness index and pulse wave velocity in healthy subjects. J. Clin. Diagn. Res. 8, BC01–BC04. 10.7860/JCDR/2014/7385.4778 PMC4225872 25386420

[B50] Mak W. Y. V. Lai W. K. C. (2015). Acute effect on arterial stiffness after performing resistance exercise by using the Valsalva Manoeuvre during exertion. Biomed. Res. Int. 2015, 1–5. 10.1155/2015/343916 26539481 PMC4619793

[B51] Mangoni A. A. Mircoli L. Giannattasio C. Ferrari A. U. Mancia G. (1996). Heart rate-dependence of arterial distensibility *in vivo*. J. Hypertens. 14, 897–901. 10.1097/00004872-199607000-00013 8818929

[B52] Mattace-Raso F. U. S. Van Der Cammen T. J. M. Hofman A. Van Popele N. M. Bos M. L. Schalekamp M. A. D. H.. (2006). Arterial stiffness and risk of coronary heart disease and stroke: the Rotterdam Study. Circulation 113, 657–663. 10.1161/CIRCULATIONAHA.105.555235 16461838

[B53] McCartney N. (1999). Acute responses to resistance training and safety. Med. Sci. Sports Exerc. 31, 31–37. 10.1097/00005768-199901000-00007 9927007

[B54] McEniery C. M. Yasmin Hall I. R. Qasem A. Wilkinson I. B. Cockcroft J. R.. (2005). Normal vascular aging: differential effects on wave reflection and aortic pulse wave velocity: the Anglo-Cardiff Collaborative Trial (ACCT). J. Am. Coll. Cardiol. 46, 1753–1760. 10.1016/j.jacc.2005.07.037 16256881

[B55] McHugh M. L. (2012). Interrater reliability: the Kappa statistic. Biochem. Med. 22, 276–282. 10.11613/BM.2012.031 23092060 PMC3900052

[B56] Melo X. Fernhall B. Santos D. A. Pinto R. Pimenta N. M. Sardinha L. B.. (2016). The acute effect of maximal exercise on central and peripheral arterial stiffness indices and hemodynamics in children and adults. Appl. Physiol. Nutr. Metab. 41, 266–276. 10.1139/apnm-2015-0204 26842667

[B57] Milatz F. Ketelhut S. Ketelhut R. G. (2015). Favorable effect of aerobic exercise on arterial pressure and aortic pulse wave velocity during stress testing. Vasa 44, 271–276. 10.1024/0301-1526/a000441 26314358

[B58] Mitchell G. F. Parise H. Benjamin E. J. Larson M. G. Keyes M. J. Vita J. A.. (2004). Changes in arterial stiffness and wave reflection with advancing age in healthy men and women: the Framingham Heart Study. Hypertension 43, 1239–1245. 10.1161/01.HYP.0000128420.01881.aa 15123572

[B59] Moore S. M. Berrones A. J. Clasey J. L. Abel M. G. Fleenor B. S. (2016). Arterial hemodynamics are impaired at rest and following acute exercise in overweight young men. Vasc. Med. 21, 497–505. 10.1177/1358863X16666692 27681487

[B60] Munir S. Jiang B. Guilcher A. Brett S. Redwood S. Marber M.. (2008). Exercise reduces arterial pressure augmentation through vasodilation of muscular arteries in humans. J. Physiol. Heart Circ. Physiol. 294, 1645–1650. 10.1152/ajpheart.01171.2007 18296568

[B61] Mutter A. F. Cooke A. B. Saleh O. Gomez Y.-H. Daskalopoulou S. S. (2016). A systematic review on the effect of acute aerobic exercise on arterial stiffness reveals a differential response in the upper and lower arterial segments. Hypertens. Res. 16, 1–27. 10.1038/hr.2016.111 27733765

[B62] Okamoto T. Masuhara M. Ikuta K. (2009). Upper but not lower limb resistance training increases arterial stiffness in humans. Eur. J. Appl. Physiol. 107, 127–134. 10.1007/s00421-009-1110-x 19533164

[B63] Perdomo S. J. Moody A. M. McCoy S. M. Barinas-Mitchell E. Jakicic J. M. Gibbs B. B. (2016). Effects on carotid-femoral pulse wave velocity 24 h post exercise in young healthy adults. Hypertens. Res. 39, 435–439. 10.1038/hr.2015.161 26763854

[B64] Pereira T. Correia C. Cardoso J. (2015). Novel methods for pulse wave velocity measurement. J. Med. Biol. Eng. 35, 555–565. 10.1007/s40846-015-0086-8 26500469 PMC4609308

[B65] Peres P. Bernardelli G. F. Mendes C. C. Fischer S. S. Servantes D. M. Medeiros W. M.. (2010). Immediate effects of submaximal effort on pulse wave velocity in patients with Marfan syndrome. Braz. J. Med. Biol. Res. 43, 397–402. 10.1590/S0100-879X2010007500021 20445953

[B66] Ranadive S. M. Fahs C. A. Yan H. Rossow L. M. Agiovlasitis S. Fernhall B. (2012). Comparison of the acute impact of maximal arm and leg aerobic exercise on arterial stiffness. Eur. J. Appl. Physiol. 112, 2631–2635. 10.1007/s00421-011-2238-z 22083536

[B67] Rehman A. Rahman A. Rasool A. (2002). Effect of angiotensin II on pulse wave velocity in humans is mediated through angiotensin II type 1 (AT(1)) receptors. J. Hum. Hypertens. 16, 261–266. 10.1038/sj.jhh.1001372 11967720

[B68] Ribeiro F. Oliveira N. L. Pires J. Alves A. J. Oliveira J. (2014). Treadmill walking with load carriage increases aortic pressure wave reflection. Rev. Port. Cardiol. 33, 425–430. 10.1016/j.repc.2013.11.012 25150924

[B69] Salvi P. Palombo C. Salvi G. M. Labat C. Parati G. Benetos A. (2013). Left ventricular ejection time, not heart rate, is an independent correlate of aortic pulse wave velocity. J. Appl. Physiol. 115, 1610–1617. 10.1152/japplphysiol.00475.2013 24052034

[B70] Sharman J. E. McEniery C. M. Campbell R. I. Coombes J. S. Wilkinson I. B. Cockcroft J. R. (2005). The effect of exercise on large artery haemodynamics in healthy young men. Eur. J. Clin. Invest. 35, 738–744. 10.1111/j.1365-2362.2005.01578.x 16313249

[B71] Sharman J. E. McEniery C. M. Campbell R. Pusalkar P. Wilkinson I. B. Coombes J. S. (2008). Nitric oxide does not significantly contribute to changes in pulse pressure amplification during light aerobic exercise. Hypertension 51, 856–861. 10.1161/HYPERTENSIONAHA.107.102558 18285615

[B72] Shirwany N. A. Zou M.-h. (2010). Arterial stiffness: a brief review. Acta Pharmacol. Sin. 31, 1267–1276. 10.1038/aps.2010.123 20802505 PMC3078647

[B73] Siasos G. Athanasiou D. Terzis G. Stasinaki A. Oikonomou E. Tsitkanou S.. (2016a). The acute impact of different types of aerobic exercise on arterial wave reflections and inflammation. Cardiology 135, 81–86. 10.1159/000445993 27287855

[B74] Siasos G. Athanasiou D. Terzis G. Stasinaki A. Oikonomou E. Tsitkanou S.. (2016b). Acute effects of different types of aerobic exercise on endothelial function and arterial stiffness. Eur. J. Prev. Cardiol. 23, 1565–1572. 10.1177/2047487316647185 27121699

[B75] Stenberg J. Astrand P. Ekblom B. Royce J. Saltin B. (1967). Hemodynamic response to work with different muscle groups, sitting and supine. J. Appl. Physiol. 22, 61–70. 10.1152/jappl.1967.22.1.61 6017655

[B76] Sterne J. A. Egger M. (2001). Funnel plots for detecting bias in meta-analysis: guidelines on choice of axis. J. Clin. Epidemiol. 54, 1046–1055. 10.1016/S0895-4356(01)00377-8 11576817

[B77] Sugawara J. Komine H. Miyazawa T. Imai T. Ogoh S. (2015). Influence of single bout of aerobic exercise on aortic pulse pressure. Eur. J. Appl. Physiol. 115, 739–746. 10.1007/s00421-014-3061-0 25428726

[B78] Sun P. Yan H. Ranadive S. M. Lane A. D. Kappus R. M. Bunsawat K.. (2015). Blood pressure changes following aerobic exercise in Caucasian and Chinese descendants. Int. J. Sports Med. 36, 189–196. 10.1055/s-0034-1390493 25329430 PMC7988840

[B79] Tai Y. L. Gerhart H. Mayo X. Kingsley J. D. (2018). Acute resistance exercise using free weights on aortic wave reflection characteristics. Clin. Physiol. Funct. Imaging 38, 145–150. 10.1111/cpf.12396 27762041

[B80] Thiebaud R. S. Fahs C. A. Rossow L. M. Loenneke J. P. Kim D. Mouser J. G.. (2016). Effects of age on arterial stiffness and central blood pressure after an acute bout of resistance exercise. Eur. J. Appl. Physiol. 116, 39–48. 10.1007/s00421-015-3242-5 26275787

[B81] Vlachopoulos C. Aznaouridis K. Stefanadis C. (2010). Prediction of cardiovascular events and all-cause mortality with arterial stiffness: a systematic review and meta-analysis. J. Am. Coll. Cardiol. 55, 1318–1327. 10.1016/j.jacc.2009.10.061 20338492

[B82] Vlachopoulos C. Dima I. Aznaouridis K. Vasiliadou C. Ioakeimidis N. Aggeli C.. (2005). Acute systemic inflammation increases arterial stiffness and decreases wave reflections in healthy individuals. Circulation 112, 2193–2200. 10.1161/CIRCULATIONAHA.105.535435 16186422

[B83] Williams M. A. Haskell W. L. Ades P. A. Amsterdam E. A. Bittner V. Franklin B. A.. (2007). Resistance exercise in individuals with and without cardiovascular disease: 2007 update: a scientific statement from the American Heart Association Council on Clinical Cardiology and Council on Nutrition, Physical Activity, and Metabolism. Circulation 116, 572–584. 10.1161/CIRCULATIONAHA.107.185214 17638929

[B84] Willich S. N. Lewis M. Lowel H. Arntz H.-R. Schubert F. Schroder R. (1993). Physical exertion as a trigger of acute myocardial infarction. N. Engl. J. Med. 329, 1684–1690. 10.1056/NEJM199312023292302 8232457

[B85] Yan H. Ranadive S. M. Heffernan K. S. Lane A. D. Kappus R. M. Cook M. D.. (2014). Hemodynamic and arterial stiffness differences between African-Americans and Caucasians after maximal exercise. Am. J. Physiol. Heart Circ. Physiol. 306, H60–68. 10.1152/ajpheart.00710.2013 24186094 PMC3920155

[B86] Yan H. Ranadive S. M. Lane-Cordova A. D. Kappus R. M. Behun M. A. Cook M. D.. (2017). The effect of acute aerobic exercise and histamine receptor blockade on arterial stiffness in African-Americans and Caucasians. J. Appl. Physiol. 122, 386–339. 10.1152/japplphysiol.01115.2015 27979988 PMC5504393

[B87] Yasmin McEniery C. M. Wallace S. Mackenzie I. S. Cockcroft J. R. Wilkinson I. B. (2004). C-reactive protein Is associated with arterial stiffness in apparently healthy individuals. Arterioscler. Thromb. Vasc. Biol. 24, 969–974. 10.1161/01.ATV.zhq0504.0173 15001456

[B88] Yoon E. Jung S. Cheun S. Oh Y. Kim S. Jae S. (2010). Effects of acute resistance exercise on arterial stiffness in young men. Korean Circ. J. 40, 16–22. 10.4070/kcj.2010.40.1.16 20111648 PMC2812793

[B89] Zieman S. J. Melenovsky V. Kass D. A. (2005). Mechanisms, pathophysiology, and therapy of arterial stiffness. Arterioscler. Thromb. Vasc. Biol. 25, 932–943. 10.1161/01.ATV.0000160548.78317.29 15731494

