# Supplementary figures and images for: Acute Effects of Exercise Mode on Arterial Stiffness and Wave Reflection in Healthy Young Adults: A Systematic Review and Meta-Analysis
Source: Front Physiol. 2018 Feb 13;9:73. doi: 10.3389/fphys.2018.00073 (PMC5816907; doi:10.3389/fphys.2018.00073)

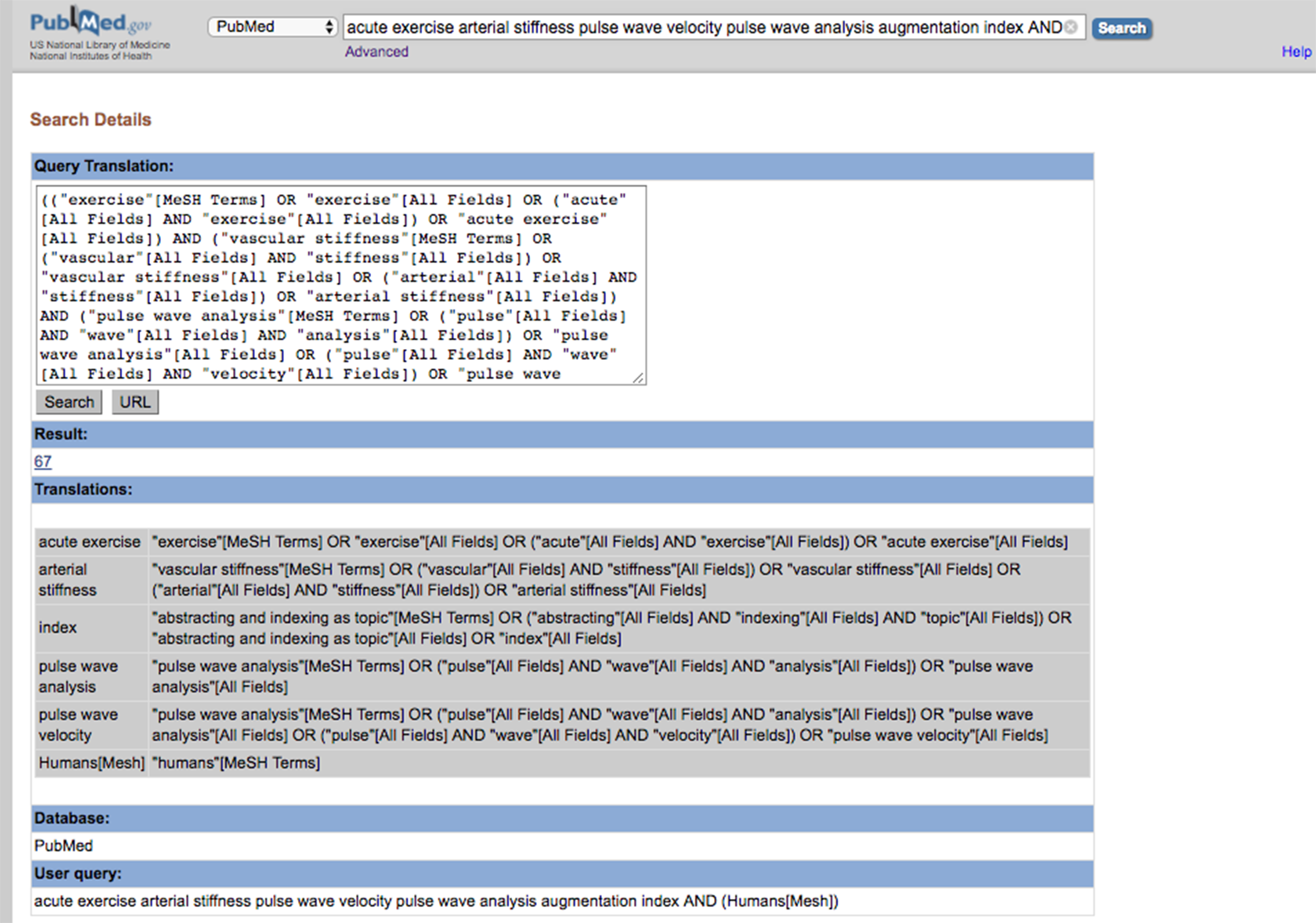

Supplement: Supplementary Figure 1 — The PubMed search strategy employed in the current review. [file Image1.TIF]

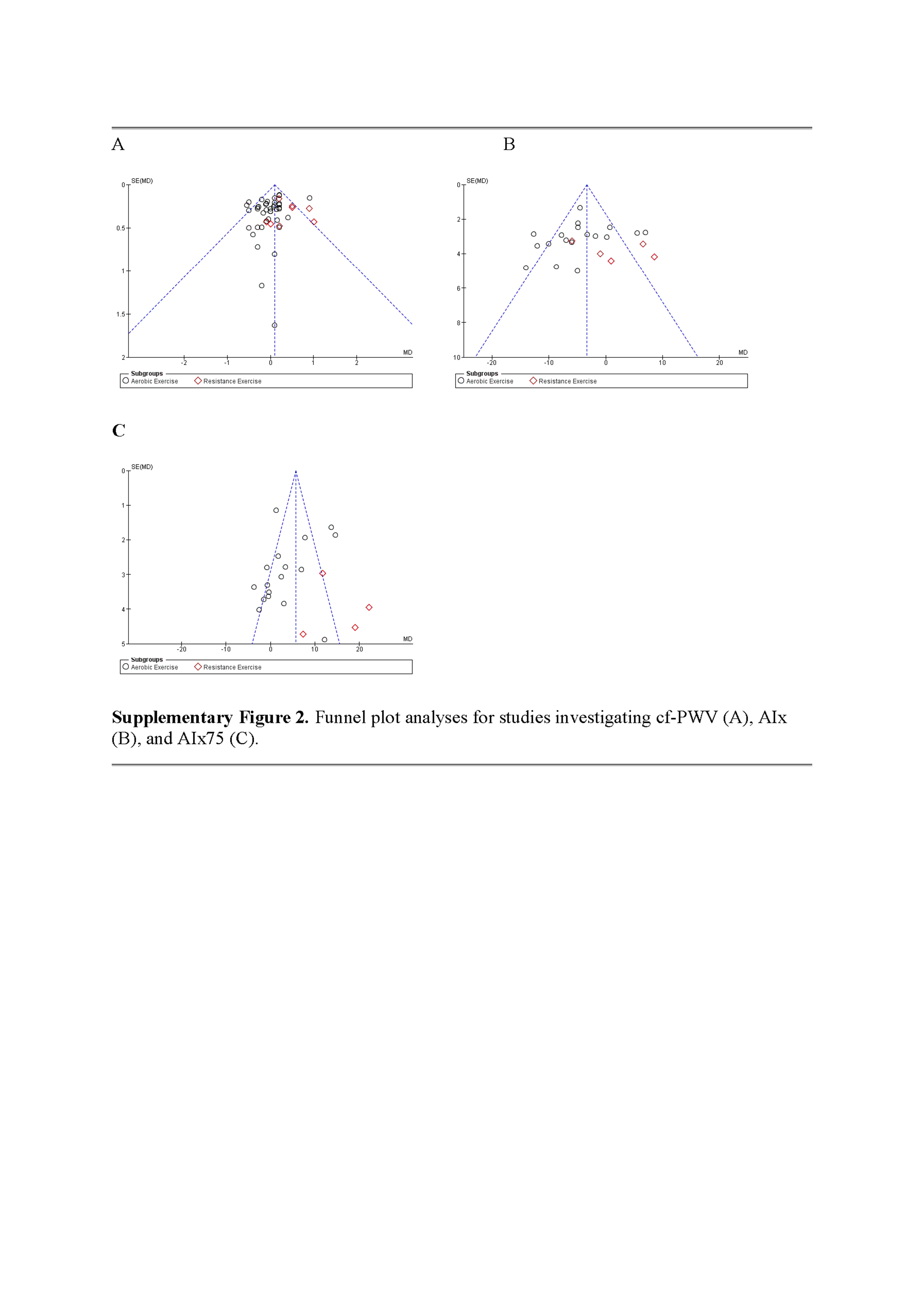

Supplement: Supplementary Figure 2 — Funnel plot analyses for studies investigating cf-PWV (A). AIx (B) and AIx75 (C). [file Image2.TIF]
