# Supplementary material for: Acute Effects of Exercise Mode on Arterial Stiffness and Wave Reflection in Healthy Young Adults: A Systematic Review and Meta-Analysis
Source: Front Physiol. 2018 Feb 13;9:73. doi: 10.3389/fphys.2018.00073 (PMC5816907; doi:10.3389/fphys.2018.00073)
Supplement: Supplementary Table 1 — Risk of Bias summary. [file Table1.pdf]

Supplementary Table 1. Risk of Bias summary

**Study/Domain**	**Selection bias (Random sequence generation)**	**Selection bias (Allocation concealment)**	**Performance bias (blinding of participants and personnel)**	**Detection bias (Blinding of outcome assessment)**	**Attrition bias (Incomplete outcome data)**	**Reporting bias (selective reporting**
Barnes et al. (2010)	🟢	🟢	🟢	🟢	🟡	🟢
Boutcher et al. (2011)	🟡	🟡	🟡	🟡	🟢	🟢
Burr et al. (2015)	🟡	🟢	🟢	🟢	🟢	🟢
Campbell et al. (2011)	🟢	🟢	🟢	🟢	🟡	🟢
Chandrakumar et al. (2015)	🟡	🟡	🟡	🟡	🟢	🟢
Collier et al. (2010)	🟢	🟢	🟢	🟢	🟡	🟢
Doonan et al. (2013)	🟡	🟡	🟡	🟡	🟢	🟢
Doonan et al. (2011)	🟢	🟢	🟢	🟢	🟡	🟢
Fahs et al. (2009)	🟢	🟢	🟢	🟢	🟢	🟢
Figueroa and Vicil (2011)	🟢	🟢	🟢	🟢	🟢	🟢
Gkaliagkousi et al. (2014)	🟡	🟡	🟡	🟡	🟢	🟢
Hanssen et al. (2015)	🟢	🟢	🟢	🟢	🟢	🟢
Heffernan et al. (2007a)	🟡	🟡	🟡	🟡	🟡	🟢
Heffernan et al. (2007b)	🟡	🟡	🟡	🟡	🟢	🟢
Heffernan et al. (2007c)	🟢	🟢	🟢	🟢	🟢	🟢
Heffernan et al. (2007d)	🟢	🟢	🟢	🟢	🟡	🟢
Heffernan et al. (2006)	🟢	🟢	🟢	🟢	🟡	🟢
Hu et al. (2013)	🟡	🟡	🟡	🟡	🟢	🟢
Hull et al. (2011)	🟡	🟡	🟡	🟡	🟢	🟢
Kingsley et al. (2016)	🟡	🟢	🟢	🟢	🟢	🟢
Kingwell et al. (1997)	🟢	🟢	🟢	🟢	🟡	🟢
Kobayashi et al. (2017)	🟢	🟢	🟢	🟢	🟡	🟢
Lane et al. (2013)	🟡	🟡	🟡	🟡	🟢	🟢

**Study/Domain**	**Selection bias (Random sequence generation)**	**Selection bias (Allocation concealment)**	**Performance bias (blinding of participants and personnel)**	**Detection bias (Blinding of outcome assessment)**	**Attrition bias (Incomplete outcome data)**	**Reporting bias (selective reporting**
Lefferts et al. (2015)	🟢	🟢	🟢	🟢	🟡	🟢
Lin *et al.* (2016	🟢	🟢	🟢	🟢	🟢	🟢
Lydakis et al. (2008)	🟡	🟡	🟡	🟢	🟢	🟢
Mak and Lai (2015)	🔴	🟢	🟢	🟢	🟡	🟢
Melo et al. (2016)	🟡	🟡	🟡	🟡	🟡	🟢
Milatz et al. (2015)	🟡	🟡	🟡	🟡	🟢	🟢
Moore et al. (2016)	🟡	🟡	🟡	🟡	🟢	🟢
Munir et al. (2008)	🟡	🟡	🟡	🟡	🟢	🟢
Perdomo et al. (2016)	🟡	🟡	🟡	🟡	🟡	🟢
Peres et al. (2010)	🟡	🟡	🟡	🟡	🟢	🟢
Ranadive et al. (2012)	🟢	🟢	🟢	🟢	🟡	🟢
Ribeiro et al. (2014)	🟢	🟢	🟢	🟢	🟡	🟢
Sharman et al. (2008)	🟢	🟢	🟢	🟢	🟢	🟢
Siasos et al. (2016a)	🟢	🟡	🟡	🟢	🟡	🟢
Siasos et al. (2016b)	🟢	🟢	🟢	🟢	🟡	🟢
Sugawara et al. (2015)	🟡	🟡	🟡	🟡	🟡	🟢
Sun et al. (2015)	🟡	🟡	🟡	🟡	🟡	🟢
Tai et al. (2018)	🟢	🟢	🟢	🟢	🟡	🟢
Thiebaud et al. (2016)	🟢	🟢	🟢	🟢	🟢	🟢
Yan et al. (2014)	🟡	🟢	🟡	🟡	🟡	🟢
Yan et al. (2017)	🟢	🟢	🟢	🟢	🟢	🟢
Yoon et al. (2010)	🟢	🟢	🟢	🟢	🟡	🟢

🟢 = low risk, 🟡 = unclear risk, 🔴 = high risk
